# Performance of different polygenic risk scores for breast cancer risk prediction: in-depth evaluations across large UK and Australian cohorts

**DOI:** 10.1038/s41431-025-02003-8

**Published:** 2026-01-13

**Authors:** Hamzeh M. Tanha, Matthew H. Law, Nathan Ingold, Catherine M. Olsen, Nirmala Pandeya, Roger L. Milne, Robert J. MacInnis, David C. Whiteman, Anne E. Cust, Julia Steinberg

**Affiliations:** 1 The Daffodil Centre, The University of Sydney, and Cancer Council NSW, Sydney, NSW Australia; 2 Department of Population Health, QIMR Berghofer Medical Research Institute, Brisbane, QLD Australia; 3 Statistical Genetics, Population Health, QIMR Berghofer Medical Research Institute, Brisbane, QLD Australia; 4 School of Biomedical Sciences, Faculty of Health, Queensland University of Technology, Brisbane, QLD Australia; 5 School of Biomedical Sciences, Faculty of Medicine, University of Queensland, Brisbane, QLD Australia; 6 Cancer Epidemiology Division, Cancer Council Victoria, Melbourne, VIC Australia; 7 Centre for Epidemiology and Biostatistics, The University of Melbourne, Parkville, VIC Australia

**Keywords:** Epidemiology, Genetics research, Breast cancer

## Abstract

Polygenic risk scores (PGS) have the potential to support enhanced, risk-based screening for breast cancer. Previous studies for many diseases found that genome-wide PGS (GW-PGS) outperform PGS derived by applying hard GWAS significance thresholds. To support future breast cancer risk predictions, we compared the predictive performance of two existing PGS (including PGS313, a leading hard-thresholding PGS) and five newly developed GW-PGS (applying different methods to recent GWAS). We evaluated the performance of PGS Z-scores and of predicted 5-year absolute breast cancer risks based on age alone or age and PGS, across three large cohorts from the UK (UK Biobank) and Australia (QSkin, Melbourne Collaborative Cohort Study). Performance was assessed using discrimination (AUC) and calibration metrics, with dedicated evaluations for European, South Asian and African genetic ancestry groups, different age groups and for UKB, by pre-baseline mammogram screening history. Z-scores from three GW-PGS (LDpred2, PRS-CS, PRS-CS_2017_) yielded improved discrimination over PGS313, especially in European and South Asian ancestry groups (AUC improvements 2–18%, *p* < 0.029). Incorporating PGS substantially improved absolute risk predictions compared to age-only models, with the strongest evidence in European-ancestry groups (AUC improvements 15–39%, *p* < 10⁻⁴) and similar trends in non-European groups. No PGS outperformed all others across all ancestry groups. Estimated relative risk for highest GW-PGS risk groups (e.g. top 5% LDpred2) was ~2.5-fold population-average risk, similar to previous estimates for individuals with pathogenic variants in *ATM* and *CHEK2* genes. These findings support the potential of PGS for risk-based breast cancer screening, noting that current GW-PGS may not substantially improve breast cancer risk predictions compared to PGS313.

## Introduction

Breast cancer is the most common cancer in women worldwide, with about 2.3 million new cases and substantial mortality each year [1]. Early detection is crucial since early-stage diagnosis improves outcomes and reduces deaths [2].

Traditional breast cancer screening relies on age-based guidelines, but this ‘one-size-fits-all’ approach ignores individual risk differences from genetics, health and behaviour [3]. In many countries, including the UK, Germany, France and Australia, breast cancer screening currently starts from age 50. Yet breast cancers in younger women are often more aggressive. Screening at age 40–49 could cut deaths but increase false positives, unnecessary biopsies and anxiety [4]. Risk-based approaches could be used to recommend different screening start ages and/or modalities, to improve early detection among high-risk groups, while reducing false-positives and over-diagnosis among low-risk women [5]. Such risk-based approaches are increasingly of interest in different countries, including the UK and Australia [6–8].

Genetic predisposition significantly influences breast cancer risk. High-penetrance pathogenic variants (e.g. in *BRCA1/2*) confer high risk, but explain few cases [9]. By contrast, polygenic risk scores (PGS) capture much of the variation in predisposition by aggregating effects of hundreds–thousands of variants discovered by genome-wide association studies (GWAS) [10]. Therefore, PGS could stratify risk to guide screening, including start age.

Some well-established and extensively validated risk tools for breast cancer already incorporate a PGS, including BOADICEA (CanRisk) [11] and IBIS [12]. Both include PGS313, developed by applying a hard *p*-value threshold to a GWAS [13]. For both risk tools, the PGS substantially improves model performance independent of other factors [14, 15].

As larger GWAS datasets and improved PGS methods emerge, comparing the performance of different breast cancer PGS is essential to optimise risk predictions. In particular, genome-wide PGS (GW-PGS) capture risk more comprehensively by including many variants, not only genome-wide significant SNPs. GW-PGS methods differ in variant selection and effect modelling, accounting for variant correlations. A Finnish study found that a GW-PGS improved risk predictions beyond clinical risk factors [16]. Notably, studies across different diseases showed that GW-PGS approaches achieve higher performance than threshold-based PGS [17–19]; thus, evaluations of GW-PGS approaches for breast cancer are needed. Such evaluations must include different populations, as PGS performance varies across populations [20, 21] and some studies showed recalibration may be needed for accuracy across risk groups and populations [14]. With GWAS consortia expanding, independent validation in cohorts outside the discovery GWAS is also needed.

This study compared published PGS with new GW-PGS for 5-year breast cancer risk prediction. We tested predictions in three large UK and Australian cohorts, one fully independent of the discovery GWAS. Analyses assessed relative and absolute risk by ancestry, age and in UK Biobank, prior screening history. To illustrate PGS potential for pre-screening risk stratification, we estimated 5-year absolute risk by age group and PGS quantile.

## Methods

A summary of cohorts and analysis steps is provided below, with detailed information in the Supplementary Information. We followed the workflow for PGS development, evaluation and downstream risk analyses as in our previous prostate cancer study [22], applying it to breast cancer GWAS data and female instead of male cohort participants.

### Cohorts

UK Biobank (UKB) [23] is a prospective cohort containing genetic and health data from >480,000 participants (>260,000 female), with the initial assessment visit (2006–2010) used as the ‘baseline’ for  risk predictions.

QSkin Sun and Health Study (QSkin) [24] is a prospective cohort from Queensland, Australia, including >16,000 participants with genetic data (>8000 female). The date of saliva collection (2014–2016) was set as the analysis ‘baseline’.

The Melbourne Collaborative Cohort Study (MCCS) [25] is a prospective cohort from Melbourne, Victoria. We analysed data for a randomly selected sub-cohort of around 4500 individuals who completed a second follow-up between 2003 and 2007, serving as the ‘baseline’ for this study (>2500 female). Genotyping in MCCS was performed for this sub-cohort and females who developed breast cancer within 5 years of the baseline.

The main outcome was diagnosis of invasive breast cancer (ICD-10 code: C50; ICD-9 code: 174), as recorded in linked population-based cancer registries (noting UK and Australian registries have high-quality data and excellent completeness [26]). Our focus was on cases diagnosed ≤5 years after the prediction baseline for each cohort.

### Individual-level quality control

Standard quality control procedures were applied to each cohort, such as excluding participants with self-reported or genetically inferred male sex, high genotype missingness or extreme heterozygosity. We also removed one randomly selected member from each pair of first- or second-degree relatives.

### Inference of genetic ancestry

The first four principal components (PCs) from the 1000 Genomes (1KG) genetic dataset [27] were derived using PLINK v1.90 [28], based on single-nucleotide polymorphisms (SNPs) pruned for linkage disequilibrium (LD). Data from the UKB, QSkin and MCCS cohorts were subsequently projected onto these PCs (Supplementary Fig. 1).

In the UKB cohort, female participants were assigned to five super-populations using k-means clustering, resulting in 212,360 females with inferred European (EUR) ancestry, 4435 South Asian (SAS), 4427 African (AFR), 1668 American and 1687 East Asian ancestry. For the QSkin and MCCS cohorts, >95% of individuals had inferred EUR ancestry (PC values within 3 standard deviations from the 1KG-EUR mean); therefore, only this ancestry group was included in further analysis (8291 QSkin and 2654 MCCS female participants).

Due to the low number of breast cancer cases (<20) among UKB participants with inferred East Asian or American ancestry, further analyses focused on the UKB-EUR, UKB-SAS, UKB-AFR, QSkin-EUR and MCCS-EUR subgroups.

### Polygenic risk scores

We included two previously published PGS: PGS313 [13] (a leading PGS included in the BOADICEA/CanRisk risk tool, based on hard thresholding of GWAS *p*-values) and PRS-CS_2017_ [29] (an existing GW-PGS), both based on a 2017 breast cancer GWAS [30].

We constructed a new GW-PGS based on a 2020 breast cancer GWAS [31], using five methods: SCT [32], LDpred2 [33], PRS-CS [34], SBayesR [35] and SBayesRC [36]. We deliberately selected methods using different approaches for LD handling, variant selection and effect estimates (see Supplementary Information). Briefly, SCT, LDpred2 and PRS-CS all use a tuning cohort (here, a randomly selected subset of ~10,000 UKB-EUR females, including 758 breast cancer cases). SCT uses penalised regression to construct a score based on multiple ‘clumping and thresholding’ scores, including LD-independent SNPs and GWAS effect estimated as weights [32]. LDpred2 explicitly models LD, with a point-normal prior for SNP effect distribution, so only SNPs with estimated non-zero effects are selected for the PGS [33]. PRS-CS also models LD, but applies a continuous shrinkage prior, so every SNP has a non-zero effect (though most are shrunk toward zero [34]). By contrast, SBayesR and SBayesRC use Bayesian mixture models that do not require a tuning cohort [35, 36]. SBayesR directly estimates the fraction of causal variants from GWAS data, with effect estimates modelled using a mix of four distributions; SBayesRC further incorporates functional annotations [37] when estimating effects. For computational feasibility, SCT, LDpred2, PRS-CS and SBayesR were restricted to 1.1 M HapMap3 variants, whereas SBayesRC used an annotation-dependent genome-wide set of 7 M variants (see Supplementary Information).

PGS for all cohorts was calculated from genotype dosage using PLINK v2.00 [28] (‘*—score’* flag), with raw values taken from the ‘*SCORE1_AVG’* output (weighted sum of dosages divided by the number of allele observations).

### Overlap of cohorts used in discovery GWAS, PGS tuning and evaluation of PGS performance

Supplementary Table 1 shows which cohorts contributed to the discovery GWAS and/or tuning steps for each PGS. MCCS participants contributed a small fraction to 2017 GWAS [30] (underlying PGS313, PRS-CS_2017_) and 2020 GWAS [31] (underlying newly generated GW-PGS), but only accounted for <2% of GWAS participants. UKB participants contributed to PRS-CS_2017_ development (tuning included ~68,000 European-ancestry females [38]). These individuals could not be excluded from our analysis (as participant IDs were unknown), which may introduce upward bias in UKB-EUR performance estimates. Moreover, a subset of 10,000 female UKB participants was used for tuning for the new SCT, LDpred2 and PRS-CS scores, but these participants were excluded from evaluation analyses.

By contrast, all evaluations in QSkin and analyses of PGS313, SBayesR and SBayesRC in UKB represented fully independent validations, with no overlap in discovery or tuning data. Likewise, evaluations conducted in non-European subgroups of UKB (SAS and AFR) were independent of both discovery GWAS and tuning datasets.

### Age-specific absolute 5-year cancer risk

Using DevCan v6.8.0, we estimated the average population absolute 5-year risk of developing breast cancer for 5-year age groups, accounting for death as a competing risk [39] (Supplementary Fig. 2). These calculations drew on population-level data, including national (for UKB) or state-specific (for QSkin and MCCS) data on breast cancer incidence and deaths, all-cause deaths and population size (details in Supplementary Information).

### Relative risks and age-PGS-specific absolute 5-year cancer risk

PGS relative risks were calculated independently for each PGS and within the five cohort subgroups, adopting the approach described by Pain et al. [40] (details see Supplementary Information). Briefly, for each PGS and cohort subgroup, the relative risk for PGS quantile *i* was defined as the probability of being a case in quantile *i* divided by the average probability of being a case. These probabilities were derived by incorporating (1) PGS Z-scores normalised within each subgroup, (2) the PGS’s predictive accuracy (the area under the ROC curve (AUC) within each subgroup) and (3) population-level disease prevalence (estimated using DevCan). The main analyses calculated relative risks across five PGS quantiles for QSkin-EUR, MCCS-EUR, UKB-SAS and UKB-AFR and twenty-five quantiles for UKB-EUR. To illustrate the risk gradient, we also estimated relative risks for 20 PGS quantiles.

We compared the estimated and observed relative risks for each PGS quantile. Observed risk was the ratio of cases in a quantile to the overall case proportion. We computed 95% confidence intervals (CIs) by estimating the standard error of the log-relative risk, deriving bounds and exponentiating them [41].

Relative risks for pathogenic variants in selected genes were taken from [42], calculated as the fraction of cases among carriers divided by the overall case fraction. For each gene, we divided cases with the variant by total carriers and then by the study-wide case proportion.

To estimate age-PGS-specific risks, we multiplied age-specific absolute risks by the relative risks associated with each participant’s PGS. For European-ancestry groups, we also estimated the 5-year absolute risk for at-risk individuals aged 40 to 75 years, stratified by five-year age groups and PGS quantiles (5 quintiles and 0–10%, 90–100%, 95–100% quantiles).

### Evaluating the performance of risk predictions

We assessed the discriminative ability of three types of predicted risk scores: (i) PGS Z-score, (ii) age-specific absolute 5-year cancer risk and (iii) age-PGS-specific absolute 5-year cancer risk. Discrimination was evaluated using the AUC, which represents the probability that a randomly chosen participant who develops cancer (a ‘case’) has a higher predicted risk than one who does not (a ‘control’). To estimate 95% CIs, we used 2000 stratified bootstrap samples. Comparisons between AUCs were conducted using two-sided DeLong tests.

We evaluated the calibration of age-specific and age-PGS-specific predicted absolute 5-year risks by calculating the ratio of expected to observed cancer cases. For MCCS, we calculated robust 95% CIs, taking into account the case-cohort design [14].

Discrimination and calibration were evaluated for each of the five cohort subgroups. For discrimination, we also completed analyses restricted to participants aged 50–69 years at baseline (common age groups included in all three cohorts), by 5-year age groups and in UKB-EUR, stratified by 5-year age group and history of self-reported breast cancer screening at baseline (Supplementary Table 2). All analyses were limited to strata with ten or more incident cancer diagnoses.

To illustrate the potential of PGS for risk stratification, we calculated the percentage of all incident cancers that occurred among participants with the highest 10%, 25% and 50% of PGS313 and LDpred2 (as an example of the GW-PGS method) in UKB-EUR and, separately, QSkin-EUR/MCCS-EUR combined. We also examined UKB participants aged 40–49 years at baseline and estimated the proportion whose 5-year age-PGS-based absolute risk exceeds 1.2% (approximate population-average 5-year breast cancer risk at age 50, the current screening start age in several countries). This included calculating the proportion of breast cancer cases diagnosed within 5 years of baseline in that age group that occurred among women with predicted risk above this threshold. For illustrative purposes, the calculation was performed using age-PGS-based absolute risks estimated using PGS313 and LDpred2.

## Results

### Participant demographics across cohort subgroups and PGS

Table 1 provides a summary of the total number of participants, age ranges and 5-year invasive breast cancer diagnoses across five cohort subgroups based on age at baseline. The 5-year incidence included 3083 cases for UKB-EUR (testing set excluding the tuning cohort), 176 for QSkin-EUR, 194 for MCCS-EUR, 43 for UKB-SAS and 27 for UKB-AFR. Details of PGS are presented in Supplementary Table 3 and Supplementary Figs. 3–5 (see also Supplementary Information text). PGS313 includes 48 indels, for which data were not available in the QSkin cohort (see Supplementary Table 3).

**Table 1:** Age distribution of participants and numbers of incident invasive breast cancers within 5 years of study baseline, by study cohort and inferred genetic ancestry subgroups.

Cohorts and ancestry groups	UKB-EUR	QSkin-EUR	MCCS-EUR	UKB-SAS	UKB-AFR
Median age at baseline (range)	58 (40–69)	60 (45–74)	63 (50–74)	53 (40–69)	51 (40–69)
**Age at baseline**	**ND/D**	**ND/D**	**ND/D**	**ND/D**	**ND/D**
40–44	20,318/181	-	-	789/6^a^	886/ ≤5^a^
45–49	27,873/367	981/17	-	816/6^a^	1062/8^a^
50–54	33,496/421	1342/27	376/34	875/7^a^	949/7^a^
55–59	39,738/575	1555/23	566/35	760/15	607/ ≤5^a^
60–64	51,773/915	1750/37	493/40	683/ ≤5^a^	487/ ≤5^a^
65–69	36,079/624	1456/45	505/48	469/ ≤5^a^	409/ ≤5^a^
70–74	-	1031/27	520/37	-	-
Total	209,277/3083	8115/176	2460/194#	4392/43	4400/27

ND: Number of females not diagnosed with breast cancer during the 5-year follow-up period after baseline; D: Number of females diagnosed with invasive breast cancer during the 5-year follow-up after baseline.

^a^These groups were not considered in the stratified analyses by age as the number of incident diagnoses is <10. Note cell sizes ≤5 were suppressed to preserve confidentiality. #MCCS had a case/sub-cohort design, leading to a higher proportion of cases among individuals with genetic data; for individuals in the sub-cohort, there were 42 incident cases within 5 years of study baseline.

### Predictive performance of PGS Z-scores

**Fig. 1:**
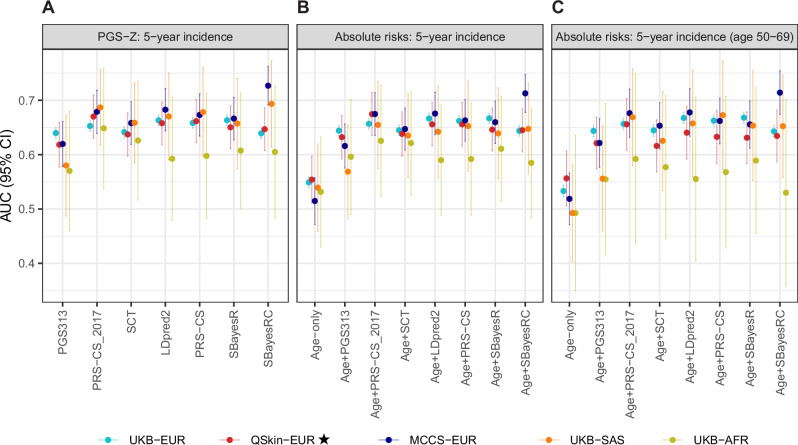
Predictive performance (AUC) of PGS Z-scores and age-specific and age-PGS-specific 5-year absolute risks. **A** AUC results based on PGS Z-scores. No single PGS had the best performance across all cohorts and ancestry groups. **B** AUC results based on predicted absolute 5-year risks. For European ancestry groups in all three cohorts and the South-Asian ancestry group in UK Biobank, 5-year risks based on age and GW-PGS performed significantly better than risks based on age alone. There was a similar pattern for the African-ancestry group in UK Biobank. Tuning data for PRS-CS_2017_ used UKB-EUR samples, overlapping with our evaluation set and potentially inflating performance in UKB-EUR results. ⋆QSkin-EUR was fully independent of both the discovery GWAS and the tuning datasets for all PGS evaluated. In addition, analyses of PGS313, SBayesR and SBayesRC in UKB were independent validations with no overlap in discovery or tuning data. Likewise, all PGS evaluations in the non-European UKB subgroups (SAS and AFR) were independent of both discovery GWAS and tuning datasets. **C** AUC results based on predicted absolute 5-year risks, among participants aged 50-69 years at baseline (common age groups included in all three cohorts). Estimates were generally similar to the main analysis of all age groups.

Figure 1A shows the AUC estimates based on within-subgroup standardised PGS (‘Z-scores’), for breast cancer diagnosis within five years of study baseline. Estimates were also similar when including both prevalent and incident cases (Supplementary Fig. 6). Overall, several GW-PGS demonstrated some improvement in AUC for European-ancestry subgroups compared to PGS313 (Supplementary Table 4). In particular, LDpred2, PRS-CS and PRS-CS_2017_ outperformed PGS313 across all three European-ancestry subgroups (AUC increase 0.013–0.063, *p* < 0.029).

In the two Australian cohorts, PGS performance was largely similar to that observed in the UKB-EUR group. One exception was a higher AUC of SBayesRC in MCCS-EUR compared to UKB-EUR and QSkin-EUR (AUC = 0.73, 95% CI:0.69–0.76 vs AUC = 0.64, 95% CI:0.63–0.65 and AUC = 65, 95% CI:0.61–0.69; *p* < 0.003).

Performance of PGS313 in UKB-SAS (AUC = 0.58, 95% CI:0.49–0.67) and UKB-AFR (AUC = 0.57, 95% CI:0.46–0.68) was slightly reduced compared to UKB-EUR (AUC = 0.64, 95% CI:0.63–0.65), but the difference was not statistically significant (*p* > 0.21). For GW-PGS, performance in UKB-SAS was similar to European-ancestry groups, with slightly lower AUC estimates in UKB-AFR. GW-PGS generally yielded higher AUC estimates than PGS313 in UKB-SAS (AUC = 0.66–0.69 vs AUC_PGS313_ = 0.58, *p *= 0.003–0.057) and UKB-AFR (AUC = 0.59–0.65 vs AUC_PGS313_ = 0.57, but *p* > 0.05). No single PGS was consistently the best across all cohorts/subgroups.

### Predictive performance of absolute 5-year risks

Age-specific risks are estimated from population-wide incidence, influenced by screening practices in each population and period. In the UK, national screening was limited to women under age 70 until 2018 and in Australia, regular invitations for women aged 70–74 were only phased in from 2013–2017. Consequently, the declines in risks at ages 70–74 estimated using population-wide data corresponding to UKB (baseline 2006–2010) and MCCS (baseline 2002–2006) are likely due to ascertainment bias rather than differences in underlying breast cancer risk. By contrast, QSkin analysis baseline (2014–2016) coincided with national screening of women up to age 74 and indeed Supplementary Fig. 2 shows higher estimated risks at ages 70–74 than at 65–69 in relevant population-wide data.

Incorporating PGS into age-specific absolute risks led to a substantial improvement in performance across European-ancestry subgroups (Fig. 1B and Supplementary Table 5), with AUC increases in UKB-EUR (0.64–0.67 for age-PGS-specific risks vs 0.55 for age-specific risks, *p* < 10^–86^), QSkin-EUR (0.63–0.67 vs 0.55, *p* < 10^–5^) and MCCS-EUR (0.62–0.71 vs 0.51, *p* < 10^–4^).

Across European-ancestry subgroups, AUC estimates were generally higher for age-PGS-specific predictions based on GW-PGS than based on PGS313 (e.g. LDpred2, PRS-CS: AUC increase 0.018–0.06), with significantly higher performance for PRS-CS_2017_ in all three European-ancestry subgroups (AUC increase 0.013–0.059, *p* < 0.01). For other GW-PGS, differences in AUC of age-PGS-specific risks compared to PGS313 did not reach statistical significance in at least one cohort (e.g. LDpred2, PRS-CS: *p *~ 0.14 in QSkin, *p* < 0.003 in UKB and MCCS; Supplementary Table 6).

Age-PGS-based absolute risks based on all examined GW-PGS had substantially higher performance than age-based absolute risks in UKB-SAS (AUC = 0.64–0.65 vs 0.54, *p* < 0.002), with similar trends for improvements observed in UKB-AFR (AUC = 0.58–0.63 vs 0.53, with *p* < 0.05 for SCT, PRS-CS_2017_, SBayesR). For PGS313, the improvement in AUC compared to age-only risks was not statistically significant in UKB-SAS or UKB-AFR (AUC increase by 0.03–0.06, *p* > 0.12).

Restriction to overlapping age at baseline (50–69) in all cohorts yielded AUC estimates similar to the main analysis of all age groups (Fig. 1C) and AUC estimates for different 5-year age groups were generally consistent (Supplementary Fig. 7).

### Predictive performance of absolute risks by pre-baseline breast cancer screening

We also evaluated AUC for UKB-EUR participants based on self-reported pre-baseline breast cancer screening (mammogram) history (Supplementary Fig. 8). Among women aged 40–44, 45–49 and 50–54 years (where sample sizes allowed assessment), there were no significant AUC differences between those with and without self-reported screening history (*p* > 0.122).

### Calibration

**Fig. 2:**
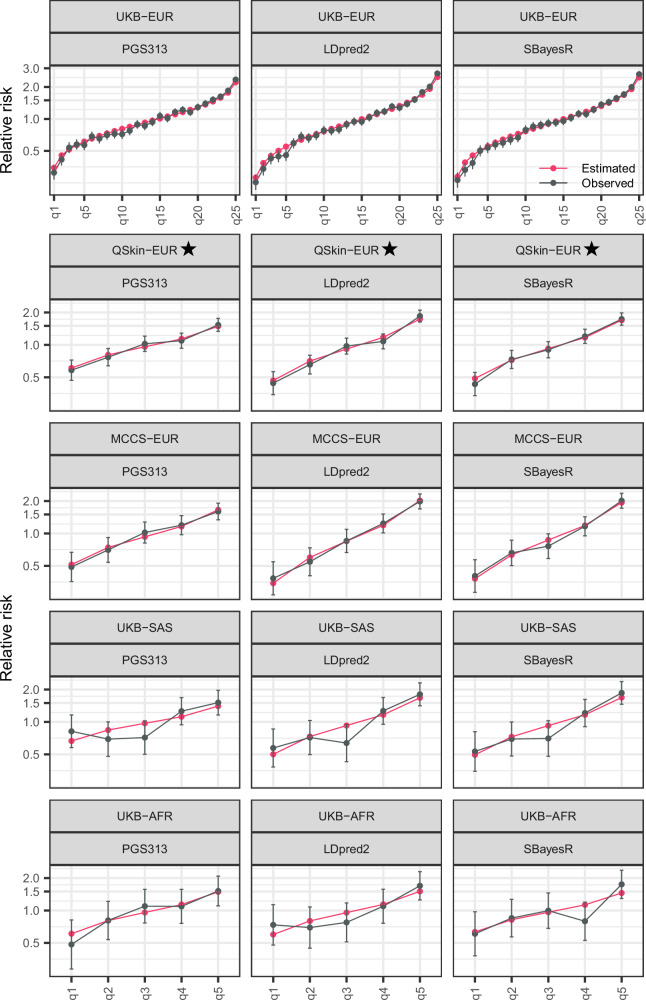
Calibration of predicted relative risks for breast cancer by PGS Z-score quantiles. The figure presents the relative risk for all breast cancer diagnoses (prevalent and incident) across PGS Z-score quantiles for the cohorts and ancestry subgroups. For UKB-EUR, 25 PGS quantiles were used, with the 13th quantile serving as the reference. For Australian cohorts and UKB non-European ancestry subgroups, five PGS quantiles were used. The red lines represent the estimated (predicted) relative risk, while the black lines indicate the observed relative risk within each PGS Z-score quantile. Bars illustrate the 95% confidence intervals for the observed relative risks. The plots illustrate that the predicted relative risks are well-calibrated, as we included cohort- and ancestry-specific AUC, as well as PGS mean and standard deviation used for standardising PGS (calculating PGS Z-scores). Results for other PGS not shown here are similar, see Supplementary Fig. 9. ⋆QSkin-EUR was fully independent of both the discovery GWAS and the tuning datasets for all PGS evaluated. In addition, analyses of PGS313 and SBayesR in UKB were independent validations with no overlap in discovery or tuning data.

The estimated and observed relative risks by PGS quantiles were closely aligned, noting estimated risks were based on subgroup-specific AUC and standardised PGS distributions (Fig. 2 and Supplementary Fig. 9).

Supplementary Figure 10 shows overall calibration results for predicted absolute risks. Age-specific absolute risks were well-calibrated in UKB-EUR (E/O = 1.00, 95% CI: 0.97–1.05), but underpredicted breast cancer incidence in QSkin-EUR (E/O = 0.79, 95% CI: 0.68–0.91) and MCCS-EUR (E/O = 0.87, 95% CI: 0.76–1.00). In UKB-SAS and UKB-AFR, age-specific predicted risks (based on population-wide data without adjustment for ancestry) significantly overestimated incidence. Age-PGS-specific risks demonstrated calibration results comparable to age-specific absolute risks in all cohort subgroups (in line with good alignment of PGS relative risks by design).

### Illustrating the potential of risk-stratification based on PGS

**Fig. 3:**
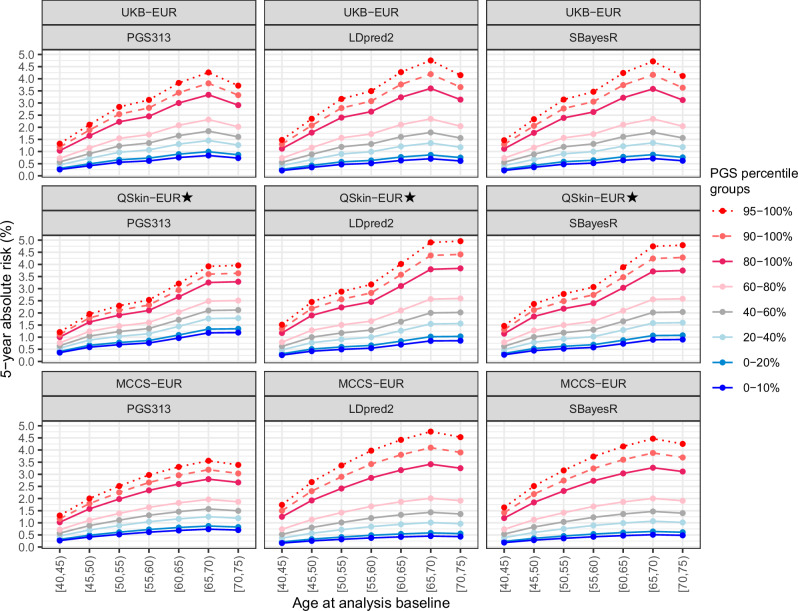
Predicted age-PGS-specific 5-year absolute risk of breast cancer by age and PGS centiles, based on relative risks estimated for participants with inferred European genetic ancestry. This illustrates marked differences between high-risk and low-risk groups at the same age. 5-year absolute risks for the 40–60% quintile (approximately equivalent to population-average risk [i.e. age-specific absolute risk]) are shown in grey. Results for other PGS not shown here are similar, see Supplementary Fig. 11. ⋆QSkin-EUR was fully independent of both the discovery GWAS and the tuning datasets for all PGS evaluated. In addition, analyses of PGS313 and SBayesR in UKB were independent validations with no overlap in discovery or tuning data. Note, the decline in risks at ages 70–74 estimated for UKB and MCCS likely reflects reduced case ascertainment due to population-wide screening practices, rather than changes in breast cancer risk.

Using relative risks for different PGS groups, the findings suggest PGS can help stratify the population into groups with different average risks (Fig. 3 and Supplementary Fig. 11). For example, across all age groups combined in the Australian cohorts, females in the top 10% of PGS313 and LDpred2 accounted for 17% and 21% of cases, respectively and those in the top 25% accounted for 41% and 49%, respectively, supporting the potential of PGS for breast cancer risk stratification. Moreover, in the Australian cohorts, females in the top 5% of PGS313 and top 10% of LDpred2 reached the population-average 5-year breast cancer risk at age 50 (~1.2%; current screening age in Australia and many other countries) about 5–10 and 10 years earlier, respectively, while those in the lowest 10% of PGS313 and 20% of LDpred2 reached this risk around 20 years later, illustrating how PGS can help inform age-specific recommendations for screening start age.

Among women aged 40–49 years at baseline in UKB-EUR, 15% and 18% had a predicted absolute 5-year risk ≥1.2% when considering age-PGS-based risks using PGS313 and LDpred2, respectively. These groups accounted for ~31% and ~39% of breast cancers diagnosed in this age band within 5 years of baseline, indicating that risk-based thresholds could identify a subset of women for earlier screening, though many cases would still occur in women with predicted risk below this threshold.

**Fig. 4:**
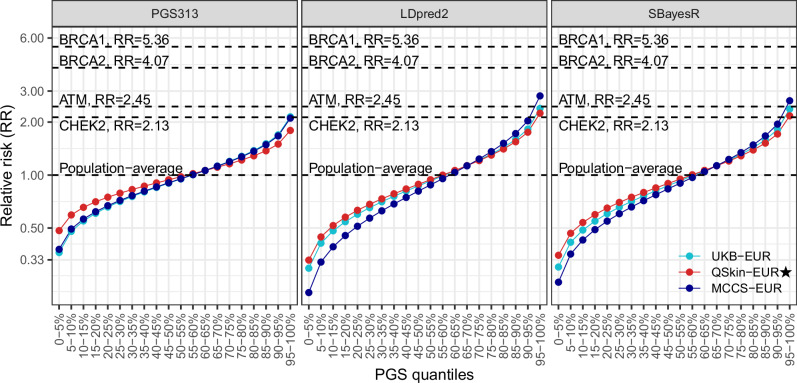
Estimated relative risks of breast cancer conferred by different levels of PGS (Z-scores) among participants with European genetic ancestry. The relative risks for pathogenic variants in specific genes were derived from breast cancer case rates reported in the study by Southey et al. [42]. Results for other PGS not shown here are similar, see Supplementary Fig. 12. ⋆QSkin-EUR was fully independent of both the discovery GWAS and the tuning datasets for all PGS evaluated. In addition, analyses of PGS313 and SBayesR in UKB were independent validations with no overlap in discovery or tuning data.

As an exploratory illustration of relative risks in European-ancestry cohorts (Fig. 4 and Supplementary Fig. 12), our estimates suggest that those with 5% highest PGS313 have a relative risk of developing breast cancer around 2-fold higher than the population average. These illustrative point estimates are higher for several GW-PGSs (as per the higher AUC of PGS Z-scores for several GW-PGS than for PGS313). In particular, for LDpred2 (higher AUC than for PGS313 among European-ancestry participants as described above) and SBayesR (no tuning data required), based on the independent QSkin data, those with 5% highest PGS have an estimated relative risk of developing breast cancer around 2.5-fold higher than the population average. These estimates are still lower than the relative risks (RR) estimated for pathogenic variants in *BRCA1* (RR = 5.36) and *BRCA2* (RR = 4.07), but comparable to the relative risks estimated for pathogenic variants in *ATM* (RR = 2.45) and *CHEK2* (RR = 2.13) [42].

## Discussion

To support future evidence-based evaluations of potential risk-based screening, this study assessed the performance of different PGS to predict breast cancer risks across three large, distinct cohorts. Specifically, we compared a range of methodologically different PGS approaches to help ensure future predictions are based on the best possible methods.

Incorporating PGS313 into age-specific absolute risks significantly improved performance in European-ancestry, but not in UKB South-Asian and African-ancestry subgroups, where smaller sample sizes yielded wider confidence intervals and limited statistical power. Nonetheless, we found that age-PGS-specific absolute risks had higher discriminative ability than age-only risks for all GW-PGS approaches included here across all European-ancestry and the UKB South-Asian-ancestry groups, and for several GW-PGS approaches, also in the UKB African-ancestry subgroup. Notably, we found that the performance of age-PGS-specific absolute risks remained consistent across age groups and there were no significant differences by pre-baseline mammogram screening in UK European-ancestry participants. The concordance in AUC estimates for European-ancestry participants in UK and Australian cohorts further supports the robustness of our performance evaluations.

With respect to selection of a best-performing PGS, there was no single approach that outperformed all others. The genome-wide methods generally yielded similar prediction performance, despite methodological differences. This could be at least partially due to the restriction to HapMap3 SNPs (except for SBayesRC), so different methods captured broadly overlapping sets of informative variants, and/or to the genetic architecture of breast cancer and the populations included here. Notably, no single method consistently outperformed the others across datasets and sensitivity analyses. Compared to PGS313 (which is incorporated in existing, validated risk tools), three GW-PGS (LDpred2, PRS-CS, PRS-CS_2017_) had significantly higher discrimination when evaluating Z-scores across all European- and UKB South-Asian ancestry subgroups, with higher AUC estimates but no statistical significance for the UKB African-ancestry subgroup. For age-PGS-specific absolute risks, only predictions based on PRS-CS_2017_ had significantly higher discrimination than predictions based on PGS313 across all European-ancestry subgroups and the UKB South-Asian-ancestry subgroup, with suggestive evidence for LDpred2 and PRS-CS (without statistically significant differences for the UKB African-ancestry subgroup). It should be noted that the ability to detect statistically significant differences in PGS performance within cohorts is limited in QSkin-EUR, MCCS-EUR and the non-European subgroups due to smaller numbers of breast cancer cases. Another limitation for the comparison is that PGS313 and PRS-CS_2017_ were developed from the 2017 breast cancer GWAS, while the five new GW-PGS were constructed using the 2020 GWAS. Ideally, utilising the same discovery GWAS would enable perfectly fair comparisons. Here, the 2020 GWAS was used to enable assessment of the best possible PGS performance. As the difference in GWAS sample size was minor (+8% cases in 2020 vs 2017) and PRS-CS_2017_ performed very similarly to PRS-CS based on the 2020 GWAS, the modest differences we observed between PGS313 and GW-PGS are unlikely to be primarily due to differences in discovery GWAS sample size. We also note that tuning data for PRS-CS_2017_ included a subset of UKB European-ancestry participants, overlapping with our UKB-EUR evaluation data. While this may inflate performance estimates in UKB-EUR, similar modest improvements of PRS-CS_2017_ over PGS313 were observed in non-European UKB subgroups and in independent Australian cohorts (QSkin, MCCS), suggesting that the UKB-EUR overlap alone does not fully account for the observed performance differences.

Consistent with past studies [43–45], performance of all PGS in our study was lower in the UKB African-ancestry  than in other subgroups, noting estimates for non-European subgroups are generally subject to higher uncertainty due to smaller participant numbers. Our results support the need for future analyses of more diverse cohorts to improve and robustly evaluate PGS performance for populations underrepresented in GWAS and PGS research. Especially for diverse cohorts, GW-PGS performance could likely be improved through larger and more diverse tuning cohorts and explicit leveraging of GWAS statistics from different ancestry groups. In the future, performance of GW-PGS might also be improved through other statistical approaches and/or wider inclusion of genetic variants (our analyses restricted PGS construction to HapMap3 variants for computational feasibility).

In practice, the advantages of potential small improvements in performance from GW-PGS (for example, here found for LDpred2, PRS-CS, PRS-CS_2017_ in European cohorts and UKB-SAS) compared to PGS313 would need to balance increased technical challenges, as GW-PGS includes a substantially higher number of variants (>500,000) than PGS313 (313 variants). Small increases in AUC would mostly impact predicted relative risk levels for highest and lowest PGS groups (as demonstrated in the illustrative analysis), so the potential population health benefits from improved performance would depend on the specific risk-based screening strategies under consideration (e.g. risk thresholds to identify women at higher risk for earlier screening).

As an illustrative example, the differences in the ages at which various PGS risk groups reach a specific risk threshold support the potential of PGS to enhance risk-based early detection, such as recommendations for screening start age. Here, despite differences in PGS approaches and age-specific risks, our illustrative results for Australia are generally aligned with analyses for other countries [46]. The thresholds we applied were chosen for illustration only; real-world screening recommendations would require formal cost-effectiveness analyses, consideration of harms and benefits and policy evaluation.

A challenge highlighted in our study is the calibration of age-specific absolute risks. For European-ancestry groups, overall calibration was accurate in UKB-EUR, with some under-prediction of risks in MCCS-EUR and QSkin-EUR (possibly reflecting higher health awareness and screening participation among cohort participants than the general population). However, the studied cohorts may be subject to a healthy volunteer bias, which could also affect calibration and generalisability. Notably, age-specific risk predictions based on population-average data strongly overestimated incidence in UKB South-Asian and African-ancestry subgroups, supporting the need to incorporate information on ancestry into absolute risks (which is now underway for CanRisk [47]).

Another key question for risk-based screening is which risk factors should be included for optimal risk assessment (balancing complexity with improvements in risk prediction). While the metrics are not directly comparable, the AUC of age-PGS313-specific 5-year risks estimated in MCCS (AUC 0.62) was similar to the previously reported C-statistic for 5-year risks based on the more comprehensive IBIS v8b and BOADICEA v5.0.0 (CanRisk) risk tools (full model included age, family history, health and behaviour factors and PGS313) in MCCS data (C-statistic 0.63-0.64) [14]. Notably, Li et al. [14] observed that 10-year risks predicted by IBIS and BOADICEA components limited to age and PGS achieved C-statistics nearly equivalent to the full models (C-statistic 0.59-0.61 for age and PGS vs. 0.62 for the full models). Other studies have also suggested that among BOADICEA model components, PGS provides the greatest contribution to risk stratification [48]. Nonetheless, inclusion of other key risk factors, such as pathogenic variants in *BRCA1*/*2* would be vital to identify a small number of individuals at the highest risk. Our analyses support previous estimates that even the highest PGS risk categories do not confer a risk increase similar to pathogenic *BRCA1*/*2* variants, but for GW-PGS, are comparable to the risk conferred through pathogenic variants in genes such as *ATM* and *CHEK2*. Overall, optimal approaches to breast cancer risk assessment are likely to be highly dependent on the context. Availability of information could be limited for some breast cancer risk factors prior to screening (e.g. no information on a person’s mammographic density being available prior to screening start age), while the assessment and use of PGS could be synergistic with future PGS assessments for different cancers and other preventable diseases [49].

This study has several limitations. Screening behaviours strongly influence breast cancer detection rates, affecting calibration; here, pre-baseline data were based on self-report (possibly including misclassification) and no screening data during follow-up periods were available. The Australian cohorts overwhelmingly included European-ancestry individuals, precluding dedicated analyses of other ancestries in Australian cohorts. The number of individuals in non-European subgroups of UKB was also relatively small; thus, validation of findings in other cohorts is required. Moreover, the studied cohorts may represent a healthy volunteer bias, which could affect generalisability. Calibration results showed that observed cases exceeded expectations in both cohorts, likely reflecting higher health awareness and likely screening participation among cohort participants, which may influence case ascertainment. Subsets of MCCS and UKB participants overlapped with GWAS discovery or PGS tuning data, which could inflate PGS performance in those cohorts. However, QSkin provided valuable independent validation. The close agreement between QSkin, MCCS and UKB results suggests a limited impact from sample overlap on the findings in this study. Another limitation is that SNP inclusion criteria differed across cohorts due to differences in QC pipelines, genotyping arrays and imputation panels. For example, QSkin imputation with HRC does not include indels present in PGS313; however, AUC estimates remained consistent with those from UKB and MCCS, suggesting minimal impact on performance.

This study also has several notable strengths. We used data from three well-established UK and Australian cohorts, allowing comparisons of PGS performance across different populations. Notably, the QSkin data did not contribute to the GWAS or PGS development, enabling independent assessments of PGS performance. Moreover, absolute risks were adjusted for competing mortality, helping to avoid overestimation of cancer risk in older individuals. Finally, relative risks for different PGS quantiles were calculated using the Pain et al. method [40], which leverages quantitative genetics theory and the liability threshold model, to map standardised PGS values onto relative risks across the population distribution.

In conclusion, our findings support the potential of PGS for risk stratification. Our study provides a side-by-side comparison of multiple GW-PGS approaches against the established PGS313 across large cohorts, with independent validation in QSkin. The results indicate that the GW-PGS examined here may yield some, but only small, performance gains compared to PGS313 and no single PGS method consistently outperformed the others. Given that our analyses relied on predominantly European-based GWAS and relatively small non-European-ancestry cohorts, future research using more diverse and larger datasets and developing novel methods may yield further improvements.

## Supplementary information

Supplemental Information

Supplementary Table 4

Supplementary Table 5

Supplementary Table 6

## Data Availability

The UK Biobank data are available from: http://www.ukbiobank.ac.uk/. The QSkin data can be made available upon request to david.whiteman@qimrberghofer.edu.au. The MCCS data can be made available on request to pedigree@cancervic.org.au. All five newly developed PGSs are deposited in the PGS Catalog [50] (https://www.pgscatalog.org), under accession codes PGS005345–PGS005349, associated with publication ID PGP000771.

## References

[1] Sung H, Ferlay J, Siegel RL, Laversanne M, Soerjomataram I, Jemal A, et al. Global Cancer Statistics 2020: GLOBOCAN Estimates of Incidence and Mortality Worldwide for 36 Cancers in 185 Countries. CA a cancer J Clin. 2021;71:209–49. 10.3322/caac.21660 33538338

[2] Ginsburg O, Yip CH, Brooks A, Cabanes A, Caleffi M, Dunstan Yataco JA, et al. Breast cancer early detection: a phased approach to implementation. Cancer. 2020;126:2379–93. 32348566 10.1002/cncr.32887 PMC7237065

[3] Pashayan N, Antoniou AC, Ivanus U, Esserman LJ, Easton DF, French D, et al. Personalized early detection and prevention of breast cancer: ENVISION consensus statement. Nat Rev Clin Oncol. 2020;17:687–705. 32555420 10.1038/s41571-020-0388-9 PMC7567644

[4] Grimm LJ, Avery CS, Hendrick E, Baker JA. Benefits and risks of mammography screening in women ages 40 to 49 Years. J Prim Care Community Health. 2022;13:21501327211058322. 35068237 10.1177/21501327211058322 PMC8796062

[5] Moorthie S, Babb de Villiers C, Burton H, Kroese M, Antoniou AC, Bhattacharjee P, et al. Towards implementation of comprehensive breast cancer risk prediction tools in health care for personalised prevention. Prev Med. 2022;159:107075. 35526672 10.1016/j.ypmed.2022.107075

[6] Harkness EF, Astley SM, Evans DG. Risk-based breast cancer screening strategies in women. Best Pract Res Clin Obstet Gynaecol. 2020;65:3–17. 31848103 10.1016/j.bpobgyn.2019.11.005

[7] McWilliams L, Evans DG, Payne K, Harrison F, Howell A, Howell SJ et al. Implementing risk-stratified breast screening in England: an agenda-setting meeting. Cancers 2022;14:4636. 10.3390/cancers14194636 PMC9563640 36230559

[8] Cancer Australia. The Australian Cancer Plan. 2023; https://www.australiancancerplan.gov.au/.

[9] Antoniou A, Pharoah PD, Narod S, Risch HA, Eyfjord JE, Hopper JL, et al. Average risks of breast and ovarian cancer associated with BRCA1 or BRCA2 mutations detected in case Series unselected for family history: a combined analysis of 22 studies. Am J Hum Genet. 2003;72:1117–30. 12677558 10.1086/375033 PMC1180265

[10] Roberts E, Howell S, Evans DG. Polygenic risk scores and breast cancer risk prediction. Breast. 2023;67:71–77. 36646003 10.1016/j.breast.2023.01.003 PMC9982311

[11] Carver T, Hartley S, Lee A, Cunningham AP, Archer S, Babb de Villiers C, et al. CanRisk Tool: a web interface for the prediction of breast and ovarian cancer risk and the likelihood of carrying genetic pathogenic variants. Cancer Epidemiol Biomarkers Prev. 2021;30:469–73. 33335023 10.1158/1055-9965.EPI-20-1319 PMC7611188

[12] Tyrer J, Duffy SW, Cuzick J. A breast cancer prediction model incorporating familial and personal risk factors. Stat Med. 2004;23:1111–30. 15057881 10.1002/sim.1668

[13] Mavaddat N, Michailidou K, Dennis J, Lush M, Fachal L, Lee A, et al. Polygenic risk scores for prediction of breast cancer and breast cancer subtypes. Am J Hum Genet. 2019;104:21–34. 30554720 10.1016/j.ajhg.2018.11.002 PMC6323553

[14] Li SX, Milne RL, Nguyen-Dumont T, Wang X, English DR, Giles GG et al. Prospective evaluation of the addition of polygenic risk scores to breast cancer risk models. JNCI Cancer Spectr. 2021;5:pkab021. 10.1093/jncics/pkab021 PMC8099999 33977228

[15] Mbuya-Bienge C, Pashayan N, Kazemali CD, Lapointe J, Simard J, Nabi H. A systematic review and critical assessment of breast cancer risk prediction tools incorporating a polygenic risk score for the general population. Cancers 2023;15:5380. 10.3390/cancers15225380 PMC10670420 38001640

[16] Mars N, Koskela JT, Ripatti P, Kiiskinen TTJ, Havulinna AS, Lindbohm JV, et al. Polygenic and clinical risk scores and their impact on age at onset and prediction of cardiometabolic diseases and common cancers. Nat Med. 2020;26:549–57. 32273609 10.1038/s41591-020-0800-0

[17] Dikilitas O, Schaid DJ, Kosel ML, Carroll RJ, Chute CG, Denny JA, et al. Predictive utility of polygenic risk scores for coronary heart disease in three major racial and ethnic groups. Am J Hum Genet. 2020;106:707–16. 32386537 10.1016/j.ajhg.2020.04.002 PMC7212267

[18] Thomas M, Sakoda LC, Hoffmeister M, Rosenthal EA, Lee JK, van Duijnhoven FJB, et al. Genome-wide modeling of polygenic risk score in colorectal cancer risk. Am J Hum Genet. 2020;107:432–44. 32758450 10.1016/j.ajhg.2020.07.006 PMC7477007

[19] Zhang YD, Hurson AN, Zhang H, Choudhury PP, Easton DF, Milne RL, et al. Assessment of polygenic architecture and risk prediction based on common variants across fourteen cancers. Nat Commun. 2020;11:3353. 32620889 10.1038/s41467-020-16483-3 PMC7335068

[20] Monti R, Eick L, Hudjashov G, Läll K, Kanoni S, Wolford BN, et al. Evaluation of polygenic scoring methods in five biobanks shows larger variation between biobanks than methods and finds benefits of ensemble learning. Am J Hum Genet. 2024;111:1431–47. 38908374 10.1016/j.ajhg.2024.06.003 PMC11267524

[21] Yiangou K, Mavaddat N, Dennis J, Zanti M, Wang Q, Bolla MK, et al. Polygenic score distribution differences across European ancestry populations: implications for breast cancer risk prediction. Breast Cancer Res. 2024;26:189. 39734228 10.1186/s13058-024-01947-x PMC11682615

[22] Tanha HM, Law MH, Ingold N, Ly P, Olsen CM, Pandeya N et al. Polygenic risk scores for prostate cancer: comparative evaluations in UK and Australian cohorts. HGG Adv. 2025:6;100477. 10.1016/j.xhgg.2025.100477 PMC12304678 40629691

[23] Bycroft C, Freeman C, Petkova D, Band G, Elliott LT, Sharp K, et al. The UK Biobank resource with deep phenotyping and genomic data. Nature. 2018;562:203–9. 30305743 10.1038/s41586-018-0579-z PMC6786975

[24] Olsen CM, Green AC, Neale RE, Webb PM, Cicero RA, Jackman LM, et al. Cohort profile: the QSkin Sun and Health study. Int J Epidemiol. 2012;41:929–929i. 22933644 10.1093/ije/dys107

[25] Milne RL, Fletcher AS, MacInnis RJ, Hodge AM, Hopkins AH, Bassett JK, et al. Cohort profile: the Melbourne Collaborative Cohort study (Health 2020). Int J Epidemiol. 2017;46:1757–1757i. 28641380 10.1093/ije/dyx085

[26] Curado M, Edwards B, Shin H, Storm H, Ferlay J, Heanue M et al: Cancer incidence in five continents, vol. IX, Vol. 160. IARC scientific publications Lyon, 2007.

[27] Abecasis GR, Auton A, Brooks LD, DePristo MA, Durbin RM, Handsaker RE, et al. An integrated map of genetic variation from 1,092 human genomes. Nature. 2012;491:56–65. 23128226 10.1038/nature11632 PMC3498066

[28] Chang CC, Chow CC, Tellier LC, Vattikuti S, Purcell SM, Lee JJ. Second-generation PLINK: rising to the challenge of larger and richer datasets. GigaScience. 2015;4:7. 25722852 10.1186/s13742-015-0047-8 PMC4342193

[29] Fritsche LG, Patil S, Beesley LJ, VandeHaar P, Salvatore M, Ma Y, et al. Cancer PRSweb: an online repository with polygenic risk scores for major cancer traits and their evaluation in two independent Biobanks. Am J Hum Genet. 2020;107:815–36. 32991828 10.1016/j.ajhg.2020.08.025 PMC7675001

[30] Michailidou K, Lindström S, Dennis J, Beesley J, Hui S, Kar S, et al. Association analysis identifies 65 new breast cancer risk loci. Nature. 2017;551:92–94. 29059683 10.1038/nature24284 PMC5798588

[31] Zhang H, Ahearn TU, Lecarpentier J, Barnes D, Beesley J, Qi G, et al. Genome-wide association study identifies 32 novel breast cancer susceptibility loci from overall and subtype-specific analyses. Nat Genet. 2020;52:572–81. 32424353 10.1038/s41588-020-0609-2 PMC7808397

[32] Privé F, Vilhjálmsson BJ, Aschard H, Blum MGB. Making the most of clumping and thresholding for polygenic scores. Am J Hum Genet. 2019;105:1213–21. 31761295 10.1016/j.ajhg.2019.11.001 PMC6904799

[33] Privé F, Arbel J, Vilhjálmsson BJ. LDpred2: better, faster, stronger. Bioinforma. 2021;36:5424–31. 10.1093/bioinformatics/btaa1029 PMC8016455 33326037

[34] Ge T, Chen CY, Ni Y, Feng YA, Smoller JW. Polygenic prediction via Bayesian regression and continuous shrinkage priors. Nat Commun. 2019;10:1776. 30992449 10.1038/s41467-019-09718-5 PMC6467998

[35] Lloyd-Jones LR, Zeng J, Sidorenko J, Yengo L, Moser G, Kemper KE, et al. Improved polygenic prediction by Bayesian multiple regression on summary statistics. Nat Commun. 2019;10:5086. 31704910 10.1038/s41467-019-12653-0 PMC6841727

[36] Zheng Z, Liu S, Sidorenko J, Wang Y, Lin T, Yengo L, et al. Leveraging functional genomic annotations and genome coverage to improve polygenic prediction of complex traits within and between ancestries. Nat Genet. 2024;56:767–77. 38689000 10.1038/s41588-024-01704-y PMC11096109

[37] Gazal S, Finucane HK, Furlotte NA, Loh PR, Palamara PF, Liu X, et al. Linkage disequilibrium-dependent architecture of human complex traits shows the action of negative selection. Nat Genet. 2017;49:1421–7. 28892061 10.1038/ng.3954 PMC6133304

[38] PGS000508 (2020). PGS Catalog. Accessed 3 June 2025. https://www.pgscatalog.org/score/PGS000508/.

[39] Fay MP, Pfeiffer R, Cronin KA, Le C, Feuer EJ. Age-conditional probabilities of developing cancer. Stat Med. 2003;22:1837–48. 12754719 10.1002/sim.1428 PMC1475950

[40] Pain O, Gillett AC, Austin JC, Folkersen L, Lewis CM. A tool for translating polygenic scores onto the absolute scale using summary statistics. Eur J Hum Genet. 2022;30:339–48. 34983942 10.1038/s41431-021-01028-z PMC8904577

[41] Morris JA, Gardner MJ. Calculating confidence intervals for relative risks (odds ratios) and standardised ratios and rates. Br Med J (Clin Res Ed). 1988;296:1313–6. 10.1136/bmj.296.6632.1313 PMC2545775 3133061

[42] Southey MC, Dowty JG, Riaz M, Steen JA, Renault AL, Tucker K, et al. Population-based estimates of breast cancer risk for carriers of pathogenic variants identified by gene-panel testing. NPJ breast cancer. 2021;7:153. 34887416 10.1038/s41523-021-00360-3 PMC8660783

[43] Ho WK, Tai MC, Dennis J, Shu X, Li J, Ho PJ, et al. Polygenic risk scores for prediction of breast cancer risk in Asian populations. Genet Med J Am Coll Med Genet. 2022;24:586–600. 10.1016/j.gim.2021.11.008 PMC7612481 34906514

[44] Ho WK, Tan MM, Mavaddat N, Tai MC, Mariapun S, Li J, et al. European polygenic risk score for prediction of breast cancer shows similar performance in Asian women. Nat Commun. 2020;11:3833. 32737321 10.1038/s41467-020-17680-w PMC7395776

[45] Du Z, Gao G, Adedokun B, Ahearn T, Lunetta KL, Zirpoli G, et al. Evaluating polygenic risk scores for breast cancer in women of African Ancestry. J Natl Cancer Inst. 2021;113:1168–76. 33769540 10.1093/jnci/djab050 PMC8418423

[46] Jermy B, Läll K, Wolford BN, Wang Y, Zguro K, Cheng Y, et al. A unified framework for estimating country-specific cumulative incidence for 18 diseases stratified by polygenic risk. Nat Commun. 2024;15:5007. 38866767 10.1038/s41467-024-48938-2 PMC11169548

[47] Ficorella L, Yang X, Mavaddat N, Carver T, Hassan H, Dennis J et al. Adapting the BOADICEA breast and ovarian cancer risk models for the ethnically diverse UK population. Br J Cancer. 2025;133:844–855. 10.1038/s41416-025-03117-y PMC12449465 40676226

[48] Yang X, Eriksson M, Czene K, Lee A, Leslie G, Lush M, et al. Prospective validation of the BOADICEA multifactorial breast cancer risk prediction model in a large prospective cohort study. J Med Genet. 2022;59:1196–205. 36162852 10.1136/jmg-2022-108806 PMC9691822

[49] Lennon NJ, Kottyan LC, Kachulis C, Abul-Husn NS, Arias J, Belbin G, et al. Selection, optimization and validation of ten chronic disease polygenic risk scores for clinical implementation in diverse US populations. Nat Med. 2024;30:480–7. 38374346 10.1038/s41591-024-02796-z PMC10878968

[50] Lambert SA, Gil L, Jupp S, Ritchie SC, Xu Y, Buniello A, et al. The Polygenic Score Catalog as an open database for reproducibility and systematic evaluation. Nat Genet. 2021;53:420–5. 33692568 10.1038/s41588-021-00783-5 PMC11165303

