# Supplementary material for: Performance of different polygenic risk scores for breast cancer risk prediction: in-depth evaluations across large UK and Australian cohorts
Source: Eur J Hum Genet. 2026 Jan 13;34(2):278–87. doi: 10.1038/s41431-025-02003-8 (PMC12858958; doi:10.1038/s41431-025-02003-8)
Supplement: Supplementary file 1 — Supplemental Information [file 41431_2025_2003_MOESM1_ESM.pdf]

Performance of different polygenic risk scores for breast cancer risk prediction: in-depth evaluations across large UK and Australian cohorts

1. Supplementary Information

The methodology used in this study closely follows that of our previous work on prostate cancer[1]. Thus, the following text is reproduced from the supplementary information of the previous study, with adjustments made to reflect the focus on female individuals in this study, and relevant methodological changes. The study Methods are detailed below, adhering to published guidelines for polygenic risk score (PGS) studies to promote reproducibility and facilitate future applications[2].

1.1. Cohorts

UK Biobank (UKB). For UKB[3], participants were genotyped using either the UKB Affymetrix Axiom array (89%) or the UK BiLEVE array (11%), with imputation performed based on the Haplotype Reference Consortium (HRC) and the combined UK10K and 1000 Genomes phase 3 (1KG) reference panels[3]. For this study, the "baseline" was defined as the date of the initial assessment visit (Data-Field 53) conducted between 2006 and 2010. Cancer type and diagnosis dates were determined through linkage to cancer registry records, including Data-Field 40006 for cancer type (ICD10), Data-Field 40013 for cancer type (ICD9), and Data-Field 40005 for cancer diagnosis date. These records were updated and verified for completeness until 31 December 2020 for participants in England, 31 December 2016 for those in Wales, and 30 November 2021 for those in Scotland.

Under Application Number 61181, we accessed epidemiological and genetic data from 488,146 UK Biobank participants, excluding individuals who had withdrawn before the study analysis began.

Furthermore, we assessed self-reported breast screening based on participants' responses to the question "Have you ever been for breast cancer screening (a mammogram)?" at the first assessment visit (Data-Field 2674). Response options included "Yes", "No", "Do not know" and "Prefer not to answer". Responses falling under "Yes" and "No" referred to "Screened" and "Unscreened", respectively (Supplementary Table 2).

QSkin Sun and Health Study (QSkin). For QSkin[4], genotyping was carried out using the Illumina Global Screening Array (San Diego, CA, USA), with imputation performed using the Haplotype Reference Consortium (HRC) reference panel[5]. In this study, the date of saliva collection (2014–2016) was considered the "baseline." Breast cancer diagnoses were obtained through linkage to the Queensland Cancer Registry, with records updated and validated for completeness up to December 2021.

Epidemiological and genetic data were obtained for 16,257 QSkin participants.

Melbourne Collaborative Cohort Study (MCCS). The MCCS[6] is a prospective cohort study, utilising a case-cohort design, based in Melbourne, Victoria, Australia. A sub-cohort of 4,735 participants was randomly selected from those who attended the second follow-up wave (2003–2007), which serves as the baseline for this study. Breast cancer diagnoses were identified through linkage to cancer registry records, with data updated and validated for completeness up to 30 June 2016.

Genotyping was conducted for sub-cohort members and additional female participants who developed breast cancer within five years of the second follow-up visit and prior to 30 June

2016, using the Illumina Infinium OncoArray-500k. Imputation of additional genetic variants was performed via the Michigan Imputation Server, utilising the 1KG data as the reference panel.

Epidemiological and genetic data were obtained for 6,096 MCCS participants.

1.2. Primary outcome

The primary outcome of interest was invasive breast cancer (ICD10 code: C50; ICD9 code: 174) diagnosed within five years of each study's baseline (5-year incident cases).

For the UKB cohort, cancer registry data were obtained from NHS England (for participants in England and Wales) and the National Records of Scotland, NHS Central Register (for participants in Scotland), with censoring dates of 31 December 2020, 31 December 2016, and 30 November 2021 for England, Wales, and Scotland, respectively. For QSkin, data were sourced from the Queensland Cancer Registry, with a censoring date of December 2021. The MCCS cohort's cancer registry data were provided by the Victorian Cancer Registry and the Australian Cancer Database, with censoring after 30 June 2016 or 10 years following the second follow-up.

Across all cohorts, cancer registry records ensured a minimum of five years of follow-up coverage.

1.3. Individual-level quality control

UKB. Exclusion criteria included discrepancies between self-reported and genetic sex, heterozygosity or missing rate outliers (Data-Field 22027), individuals with relatedness issues (Data-Field 22021, "Participant excluded from kinship inference process" and "Ten or more third-degree relatives identified"), and participants who had withdrawn informed consent. Additionally, one individual from each pair of first- and second-degree relatives was randomly excluded based on relatedness kinship coefficients (KING kinship > 0.0884)[7], as pre-calculated by UKB.

A tuning set of approximately 10,000 females (758 breast cancer cases) with inferred European ancestry ("10KUKB") was selected for fine-tuning the development of new polygenic risk scores (PGS) using SCT, LDpred2, and PRS-CS (see below). The remaining females were assigned to the testing set, excluding those with prevalent breast cancer (diagnosed before baseline). Females aged <40 or ≥70 years at baseline were also excluded from the testing set.

QSkin. Females with prevalent breast cancer diagnosed prior to baseline and those aged <45 or ≥75 years at baseline were excluded.

MCCS. Exclusion criteria included mismatches between self-reported and genetic sex, heterozygosity outliers (±3 standard deviations from the mean of F coefficient estimates calculated using the PLINK v2.0 –het flag), and one individual randomly removed from each pair of first- and second-degree relatives identified through KING kinship coefficients (KING kinship > 0.0884)[7], computed with PLINK v2.0. Additionally, females with prevalent breast cancer diagnosed prior to baseline and those aged <50 and ≥75 years at baseline were excluded.

1.4. Ancestry Determination

The first four principal components (PC1–PC4) were derived from the 1000 Genomes (1KG)[8] dataset using PLINK v1.90 (www.cog-genomics.org/plink/1.9/) [9]. This process was based on linkage disequilibrium (LD)-pruned single nucleotide polymorphisms (SNPs) (500 kb window, $r^2 < 0.1$), minor allele frequency (MAF) > 0.05, missingness < 0.1, and SNPs meeting Hardy-Weinberg equilibrium criteria ($p > 1 \times 10^{-6}$). Data from the UKB, QSkin, and MCCS cohorts were subsequently projected onto these principal components using PLINK v1.90.

In the UK Biobank cohort, participants were assigned to five super-populations (European [EUR], African [AFR], American, East Asian, and South Asian [SAS]) through k-means clustering with five clusters. This was implemented using the "kmeans()" function in R with default parameters and the Hartigan and Wong algorithm[10]. Due to the small number of breast cancer cases (<18 5-year incidence) in the American and East Asian groups, these populations were not included in further analyses.

For the QSkin and MCCS cohorts, over 95% of participants were inferred to have European (EUR) ancestry, as determined by principal component (PC) values within ±3 standard deviations of the 1KG EUR cluster. Consequently, only the EUR ancestry group was analysed further in these cohorts.

1.5. Polygenic risk scores (PGS)

Published PGS. We included two previously published PGS: PGS313[11] (a leading PGS included in the BOADICEA/CanRisk risk tool) and PRS-CS_{2017}[12] (an existing PGS including information across the genome sourced from the PGS Catalog [PGS000508]), both based on a 2017 breast cancer GWAS[13]. For the UKB and MCCS cohorts, we aimed to include as many genetic variants as possible from these existing PGSs and, therefore, did not apply exclusions based on minor allele frequency (MAF) or imputation quality.

In the QSkin cohort, an established dataset was used that included only SNPs meeting the following criteria: MAF > 0.005, MAF < 0.45 for A/T and G/C SNPs, and an imputation quality (INFO score) > 0.6.

Newly generated PGS. We constructed five new PGS based a 2020 breast cancer GWAS[14] using five methods: SCT[15], LDpred2[16], PRS-CS[17], SBayesR[18], and SBayesRC[19] by using the settings recommended by developers. For developing new polygenic risk scores (PGSs), except for SBayesRC, we included non-palindromic SNPs from HapMap3 phase 3[20] with minor allele frequency (MAF) > 0.005 and imputation quality (INFO) > 0.4, based on UKB genetic data. For SBayesRC, SNP selection focused on those present in the functional annotation file.

SCT combines multiple clumping and thresholding (C+T) scores into a single predictive score by optimising their linear combination rather than relying on a single set of hyperparameters. The optimal combination was identified using penalized regression (k=10), achieving the highest correlation with breast cancer in the UKB tuning data. We note that Mavaddat et al.[11] also developed a penalised regression model based on 3,820 variants, conceptually similar to our SCT approach, which combines multiple clumping-and-thresholding scores. In our analyses, SCT achieved slightly better performance than PGS313, consistent with the findings reported by Mavaddat et al[11].

LDpred2 estimates the posterior mean effect size of each SNP while accounting for the effect of correlated SNPs. It employs a point-normal prior, including only a subset of SNPs with non-zero estimated effects in the PGS. Three key hyperparameters are involved: the fraction of causal SNPs, SNP-based heritability, and sparsity (True or False). Settings were optimised to maximise the area under the receiver operating characteristic curve (AUC) in the UKB tuning data, resulting in a fraction of causal SNPs set at 0.01, SNP-based heritability at 0.0861, and no sparsity, yielding an AUC of 0.65.

PRS-CS assumes a continuous shrinkage prior on SNP effects, with a global scaling parameter (ϕ) applied uniformly across all SNPs. A value of ϕ = 0.0001 produced the best predictive performance in the UKB tuning dataset (AUC = 0.65).

SBayesR adjusts GWAS SNP effect estimates using a Bayesian approach, modelling standardised SNP effects as drawn from a mixture of four distributions with scaling factors (γ) for the variance of each mixture component set at 0, 0.01, 0.1, and 1. This approach eliminates the need for a tuning sample by directly estimating the fraction of causal SNPs from GWAS summary statistics.

SBayesRC extends SBayesR by integrating functional annotations. It employs a multicomponent annotation-dependent mixture prior to model the distribution of SNP effects, allowing simultaneous estimation of annotation parameters and SNP effects across the genome. Per-SNP functional annotations were obtained in formatted file from https://gctbhub.cloud.edu.au/software/gctb, derived from the S-LDSC Baseline LDv2.2[21] framework, which includes 96 genomic features capturing functional, LD-related, and evolutionary characteristics such as coding regions, histone marks, recombination rate, background selection, and MAF-adjusted LD and allele age. These annotations were used to inform SNP-specific prior variances, allowing SBayesRC to account for the enrichment of heritability in functionally active or evolutionarily constrained regions.

When required, European ancestry LD reference panels provided by method developers were used, as the majority of GWAS participants in the 2020 GWAS were of European ancestry.

To characterise the composition of the polygenic scores, we examined the distribution of variants across chromosomes and minor allele frequency (MAF) bins, and the quantiles of assigned effect size weights. For each PGS, we used the variant lists and effect size weights provided by the corresponding method. We calculated the proportion of variants mapping to each chromosome and stratified variants into predefined MAF bins [0–0.01), [0.01–0.05), [0.05–0.1), [0.1–0.5]; we also divided effect size weights into deciles to represent weight quantiles. These distributions were summarised and plotted to compare variant representation across methods (Supplementary Figure 3).

For all cohorts, PGS for each participant were estimated using PLINK v2.00[9] --score applied to genotype dosages. We used the *SCORE1_AVG* output, which divides the weighted sum of effect-allele dosages by the number of allele observations. This choice ensures comparability of raw distributions across cohorts with differing SNP coverage. “Raw PGS” values shown in Supplementary Figure 4 correspond to SCORE1_AVG before standardisation. For all performance analyses, scores were standardised to Z-scores within cohort and ancestry group.

To assess the similarity between PGS derived from different methods, we computed pairwise Pearson correlation coefficients (r) between PGS raw values within each cohort ancestry subgroup (UKB-EUR, QSkin-EUR, MCCS-EUR, UKB-SAS, and UKB-AFR). Correlation matrices were generated using the corrplot package in R. These correlations reflect the

overall high concordance of genetic risk ranking among individuals across PGS methods (Supplementary Figure 5).

1.6. Age-specific absolute risk

The DevCan v6.8.0 software was used to calculate population-average age-specific absolute 5-year risk of developing breast cancer, accounting for death as a competing risk[22], by 5-year age groups. These calculations incorporated population-wide data on breast cancer incidence, breast cancer mortality, all-cause mortality, and population size.

For the UKB cohort, data from England, Wales, and Scotland spanning 2006–2010 were used, corresponding to the study baseline. In Scotland, breast cancer incidence data were sourced from Public Health Scotland (https://www.publichealthscotland.scot/), and mortality data came from the National Records of Scotland (https://www.nrscotland.gov.uk/). In Wales, breast cancer incidence data were obtained from Public Health Wales (https://phw.nhs.wales/). For England, breast cancer incidence, mortality for England and Wales, and population estimates for all three regions were acquired from the Office for National Statistics (https://www.ons.gov.uk/).

For the QSkin cohort, data from Queensland (2012–2016) were used, and for the MCCS cohort, data from Victoria (2002–2006) were utilised, aligning with their respective study baseline periods. In Queensland, breast cancer incidence and mortality data were requested from Cancer Alliance Queensland (https://canceralliance qld.health.qld.gov.au/). In Victoria, breast cancer incidence and mortality data were sourced from the Victorian Cancer Registry (https://www.cancervic.org.au/research/vcr). All-cause mortality data for both regions were obtained from the National Mortality Database (https://www.aihw.gov.au/about-our-data/our-data-collections/national-mortality-database), while population estimates were sourced from the Australian Bureau of Statistics (https://www.abs.gov.au/).

1.7. Detailed description of the approach for calculation of PGS-based lifetime risk

The method of Pain et al.[23] allows to estimate absolute lifetime risks of disease by quantile of the PGS distribution, based on key statistics for
- discriminative performance of a polygenic score (PGS), expressed as the area under the ROC curve (AUC);
- mean and standard deviation of the PGS (required to convert PGS raw values to Z-score);
- disease prevalence.

The procedure involves several conceptual steps and relies on distributional assumptions of PGS that are supported by quantitative genetics. The following steps 1-4 summarise calculations as described in Pain et al.[23]

Step 1. Linking AUC to separation between cases and controls.

The AUC summarises the ability of the PGS to discriminate between cases and controls. Under the assumption that case and control PGS values follow normal distributions with equal variance, the AUC can be expressed as a monotonic function of the difference in their means. This difference, Cohen's d, therefore, provides a convenient parameterisation of the separation between case and control distributions and can be calculated directly from the observed AUC as:

$$d \approx \sqrt{2}\Phi^{-1}[AUC]$$

Step 2. Defining case, control, and population distributions.

Given disease prevalence K, the overall distribution of the PGS in the study population is modelled as a mixture of the case and control distributions, with mixture weights equal to K and $(1-K)$, respectively, with the probability density function as:

$$f_{X_i}(x_i) = K f_{X_i|Y_i}(x_i|1) + (1-K) f_{X_i|Y_i}(x_i|0)$$

This mixture distribution fully characterises the unconditional distribution of the PGS in the population, while the conditional distributions of cases and controls are centred at means separated by *d* but share equal variance. These assumptions mirror those in the classical liability-threshold model of disease, where genetic risk is normally distributed in the population with different means in cases and controls.

Step 3. Defining quantile cut points.

To evaluate risk by strata of the PGS, the method divides the population distribution into equal-sized quantiles (for example, deciles). The cut points are obtained by solving for the PGS values that correspond to the required cumulative probabilities under the mixture distribution. As there is no closed-form solution, these thresholds are found using standard numerical root-finding algorithm (the uniroot function in R).

Briefly, given the quantile probability p_q, disease prevalence K, and standardized mean difference d, the corresponding polygenic score threshold tq can be obtained by solving the following equation:

$$K\Phi\left[t_q - d\right] + (1-K)\Phi\left[t_q\right] - p_q = 0$$

Φ denotes the cumulative distribution function of the standard normal distribution.

Step 4. Calculating absolute lifetime risk within quantiles.

For each quantile interval, the conditional probability of being a case is computed. This involves evaluating the probability that a case falls within the interval, weighting by disease prevalence, and dividing by the unconditional probability of belonging to that interval as:

$$p\left(Y_i = 1 \middle| t_{q-1} < X_i < t_q\right) = nK\left(\Phi\left[t_q - d\right] - \Phi\left[t_{q-1} - d\right]\right)$$

Because quantiles are defined to contain equal probability mass in the population distribution, the denominator is constant across all groups. The resulting probabilities represent the absolute lifetime risk of disease for each PGS quantile.

Assumptions and theoretical underpinnings.

This approach rests on the assumptions of normally distributed PGS in cases and controls, equal variance between groups, and a known disease prevalence. These assumptions are consistent with the quantitative genetics framework in which polygenic risk is modelled as a normally distributed liability. By combining AUC with prevalence, the method leverages summary-level performance metrics of the PGS to approximate risk stratification in the absence of individual-level genotype and phenotype data.

1.8. Incorporating PGS into age-specific absolute risk

The absolute lifetime risks (probability of being a case) estimated using the Pain et al.[23] method (explained above) were converted to relative risks for each PGS Z-score quantile, where relative risk was defined as the ratio of the absolute lifetime risk in each quantile to the mean absolute risk across all quantiles.

Age- and PGS-specific absolute risks were then obtained by multiplying these relative risks by age-specific absolute risks (5-year risk) estimated using from population-wide data.

Population prevalence was estimated as lifetime risk estimates adjusted for competing mortality, derived with DevCan.

PGS Z-scores and AUC values were computed separately within each of five ancestry-specific subgroups. To ensure representative population-wide Z-score distributions, individuals with prevalent breast cancer were retained, allowing more appropriate estimation of the mean and standard deviation of the PGS in the relevant population. The AUC was adapted to better represent lifetime breast cancer diagnoses through two modifications: (i) prevalent cases were retained, and (ii) cases were defined as either a breast cancer diagnosis at baseline or any diagnosis occurring during follow-up, rather than restricting to 5-year incident cases. The lifetime AUC estimate used here is therefore an approximation, based on inclusion of all pre-baseline and post-baseline diagnoses. Conceptually, this differs from the study's primary focus, which is restricted to incident cases within 5 years of baseline. However, the relevant AUC estimates were highly similar within each cohort (Figure 1a and Supplementary Figure 6). Because approximation of the lifetime AUC could potentially affect relative risk estimates, we conducted validation analyses. Specifically, we compared relative risks derived from this method against those estimated directly for 5-year incidence (the primary outcome). We again found strong similarity of estimates (Figure 2 and Supplementary Figure 8), supporting the validity of the PGS-based relative risk estimates in the primary analyses.

1.9. Evaluating the performance of risk predictions

We evaluated the discrimination of three predicted risk scores: (i) the PGS Z-score, (ii) age-specific absolute 5-year cancer risk, and (iii) age-PGS-specific absolute 5-year cancer risk. Discrimination was measured using the AUC, which represents the probability that the predicted risk is higher for a randomly selected "case" (a participant who develops cancer) than for a "control" (a participant who does not). An AUC of 0.5 indicates no discriminatory ability (random ranking), while an AUC of 1.0 represents perfect discrimination. AUC estimates, along with 95% confidence intervals (CIs), were calculated using the "pROC" R package with 2,000 stratified bootstrap samples (via the "ci.auc" function). Comparisons between AUCs for age-specific and age-plus-PGS-specific absolute risks were conducted using the two-sided DeLong test for paired AUC curves, implemented with the "roc.test" function in the "pROC" package.

Calibration of the age-specific and age-PGS-specific absolute 5-year risk predictions was assessed by calculating the ratio of expected (E) to observed (O) cancer cases. Robust 95% CIs were computed for the MCCS cohort to account for its case-cohort design[24].

2. Supplementary Figures

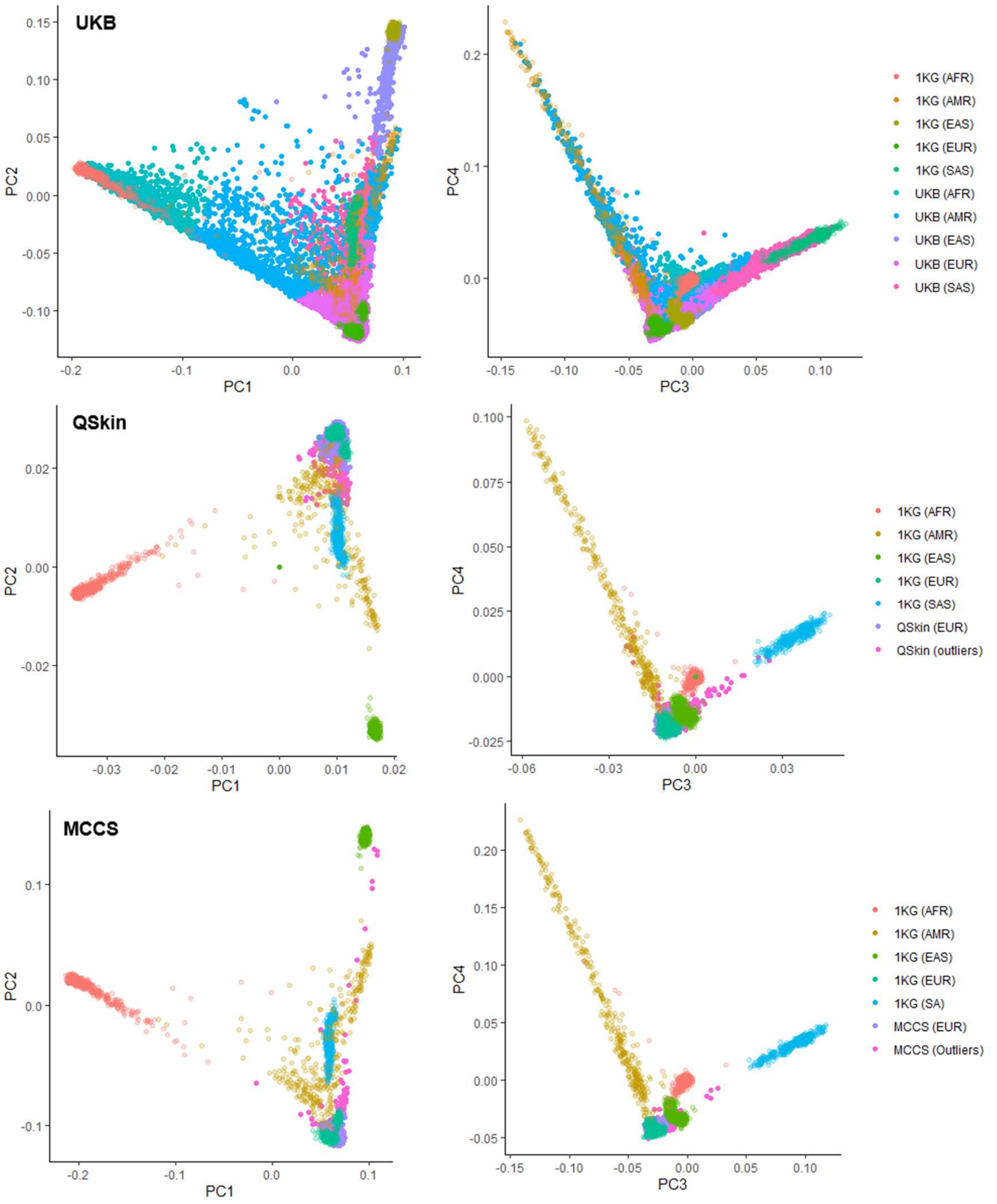

Supplementary Figure 1 – Illustration of inferred genetic ancestry, through projection onto principal components (PCs) determined from 1000 Genomes (1KG) reference data. The figure shows the projection onto the first four principal components (PC1–PC4) for each of the three major cohorts (UKB, QSkin, and MCCS). Different colours represent various ancestry groups, including African (AFR), American (AMR), East Asian (EAS), European (EUR), and South Asian (SAS) populations, highlighting clustering patterns within these cohorts. Following genetic ancestry inference as described in the Methods and Supplementary Information, the study analysed five cohort ancestry groups: UKB-EUR, UKB-SAS, UKB-AFR, QSkin-EUR, and MCCS-EUR.

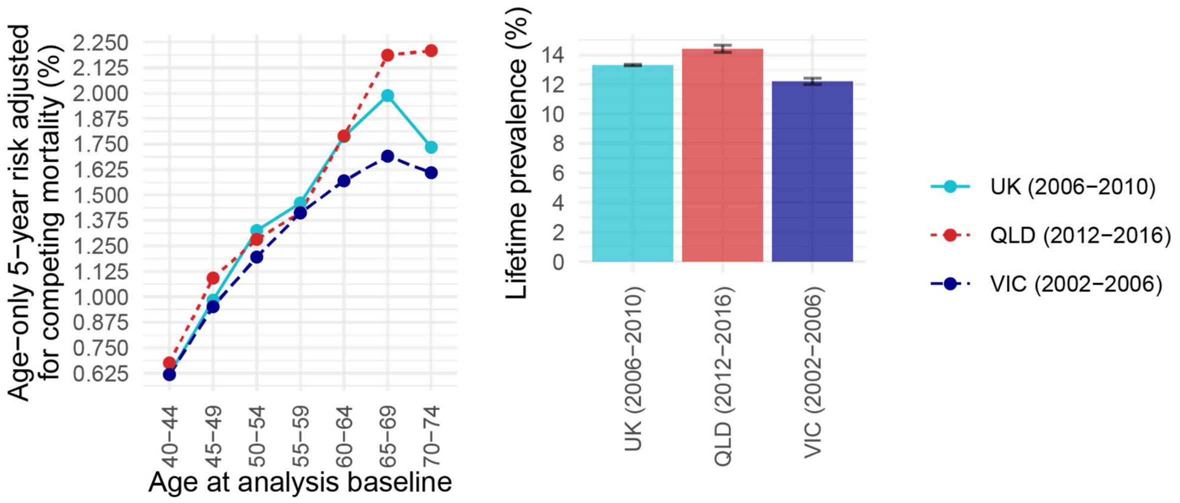

Supplementary Figure 2 – Estimated age-specific absolute risks of breast cancer. The left panel displays age-specific absolute breast cancer risks across different age groups for the UK (UKB cohort), Queensland (QLD, QSkin cohort), and Victoria (VIC, MCCS cohort), based on data corresponding to the risk prediction baseline for the relevant cohorts. The right panel shows the lifetime risk (prevalence) for breast cancer across the three cohorts (UKB, QSkin, MCCS). These estimates were calculated by DevCan and adjusted for competing mortality. The data highlights the increasing incidence of breast cancer with age and regional variations in breast cancer risks. Note, the declines in estimated age-specific risks at ages 70–74 vs 65-69 estimated for UKB likely reflects reduced case ascertainment due to screening practice, rather than changes in true underlying biological breast cancer risk. Importantly, all analyses of PGS performance in UKB in our study only included participants age <70 at baseline, so would not be affected by this artefact.

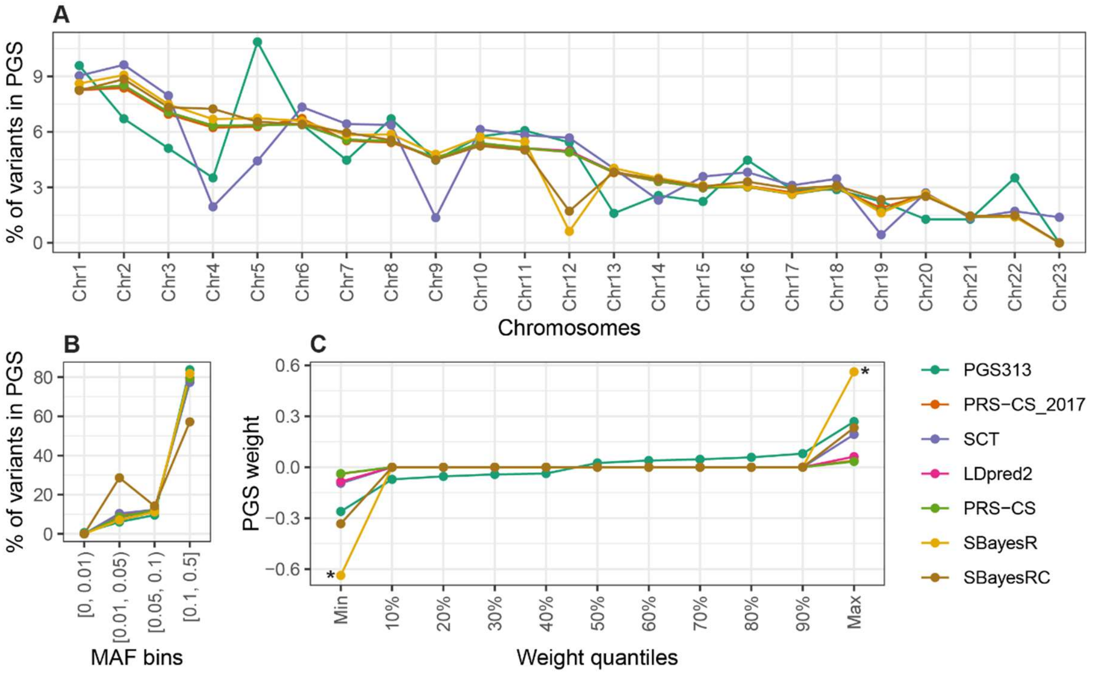

Supplementary Figure 3 – Composition of different PGS. A) Proportion of variants contributing to each PGS, for each chromosome. **B)** Distribution of variants across minor allele frequency (MAF) bins for each PGS. **C)** Distribution of variant weights for variants included in each PGS. Asterisks indicate two variants with relatively large effect sizes in SBayesR: rs7074164 (Chr10:64,564,227; G allele weight = 0.562) and rs9971363 (Chr10:64,563,702; G allele weight = –0.638). These variants are 525 bp apart and in linkage disequilibrium (D′ = 1, R^2=0.4, $p < 0.0001$ for 1000 Genomes Europeans)[25]. The predominant haplotypes for the two variants are T_A (frequency = 0.846) and G_G (frequency = 0.069), together accounting for 91.5% of haplotypes (in 1000 Genomes Europeans); these two haplotypes yield effectively balanced combined weights of approximately ±0.08, illustrating how strong local LD can produce apparent extreme effects that largely cancel in aggregate.

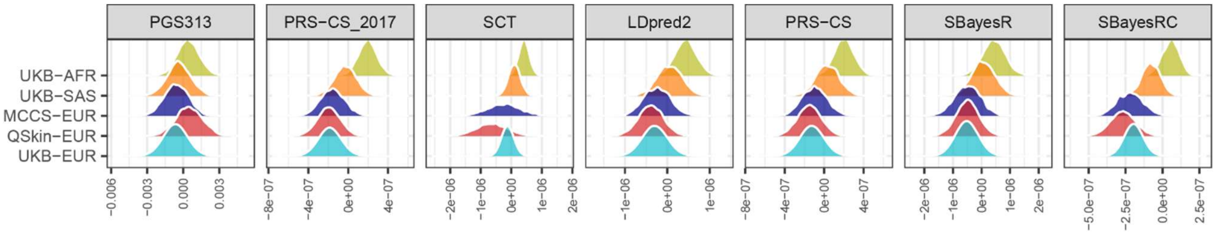

Supplementary Figure 4 - PGS distribution across cohorts and ancestry subgroups. The figure illustrates the distribution of raw PGS across five cohort ancestry groups (UKB-EUR, UKB-SAS, UKB-AFR, QSkin-EUR, and MCCS-EUR) for two existing and five newly generated PGS. The raw scores represent SCORE1_AVG estimates calculated by PLINK before standardisation. The density plots highlight differences in PGS distributions between different ancestries. Raw PGS score distributions, including mean and standard deviation before conversion to Z-scores, show consistency among European ancestry cohorts but are lower in African-ancestry and South-Asian-ancestry subgroups compared to European-ancestry subgroups, particularly for genome-wide PGS that include thousands of variants.

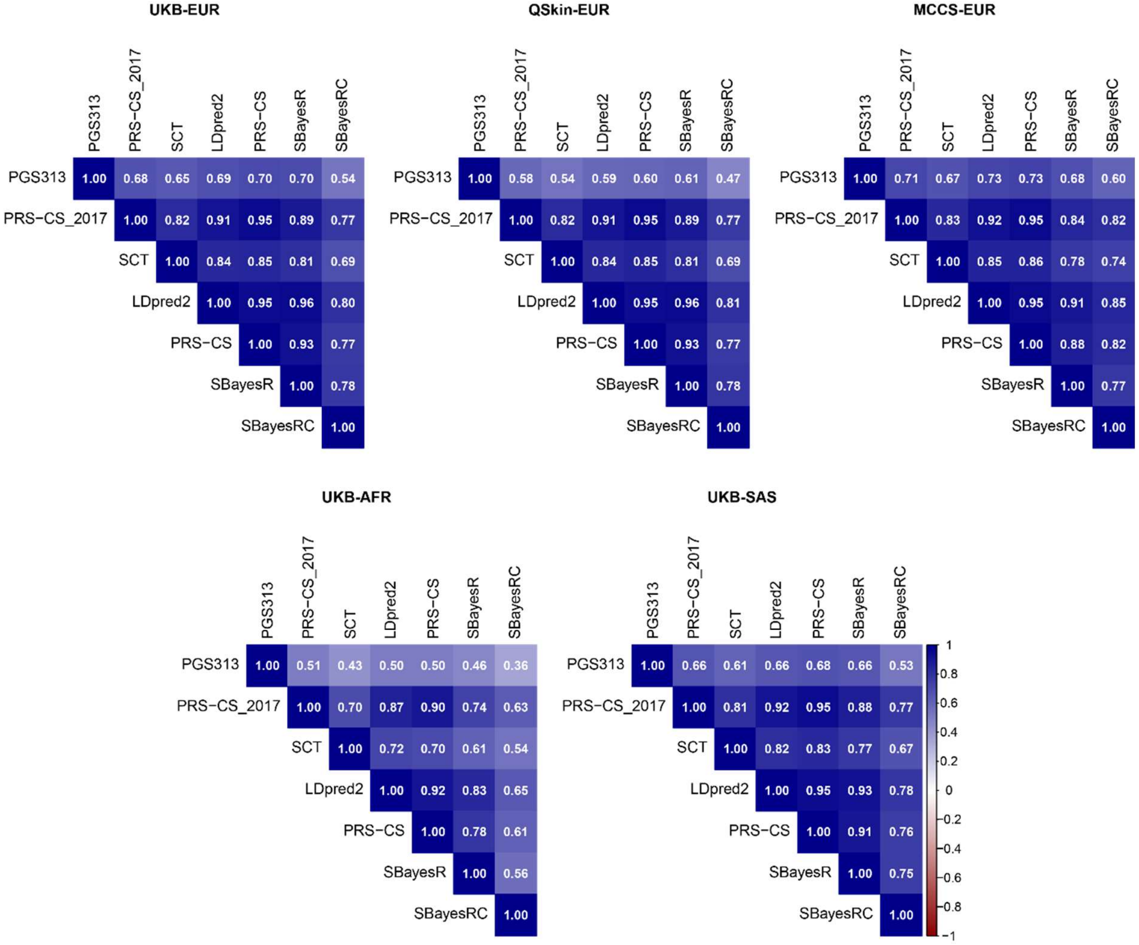

Supplementary Figure 5 - Heatmaps showing pairwise Pearson correlations between raw PGS values across individuals within each ancestry-specific cohort. Each panel corresponds to a cohort subgroup (UKB-EUR, QSkin-EUR, MCCS-EUR, UKB-SAS, and UKB-AFR). The colour scale represents the correlation coefficient (*r*). Genome-wide PGS (LDpred2, PRS-CS, SBayesR, SBayesRC, SCT, PRS-CS$_{2017}$) showed medium to strong inter-correlation, reflecting convergence in the genetic signal captured across approaches, with some differences likely due to variant selection and different modelling of LD and effect estimates. Correlations involving PGS313 were lower compared to genome-wide PGS.

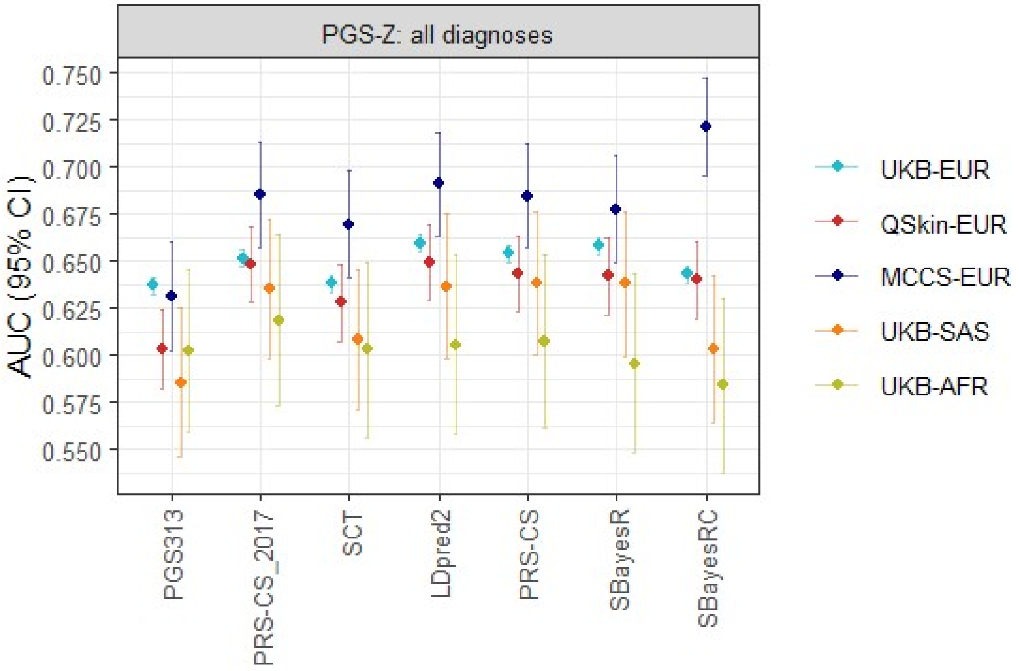

Supplementary Figure 6 - Predictive performance (AUC) of standardised PGS (PGS Z-scores) across cohort ancestry groups. The figure presents the AUC results including prevalent cases, and assessing discrimination across prevalent and all incident cases (without limitation to <5 years after baseline), for all five cohort and ancestry subgroups (UKB-EUR, QSkin EUR, MCCS EUR, UKB-SAS, and UKB AFR). These AUC estimates were used in calculating PGS relative risks.

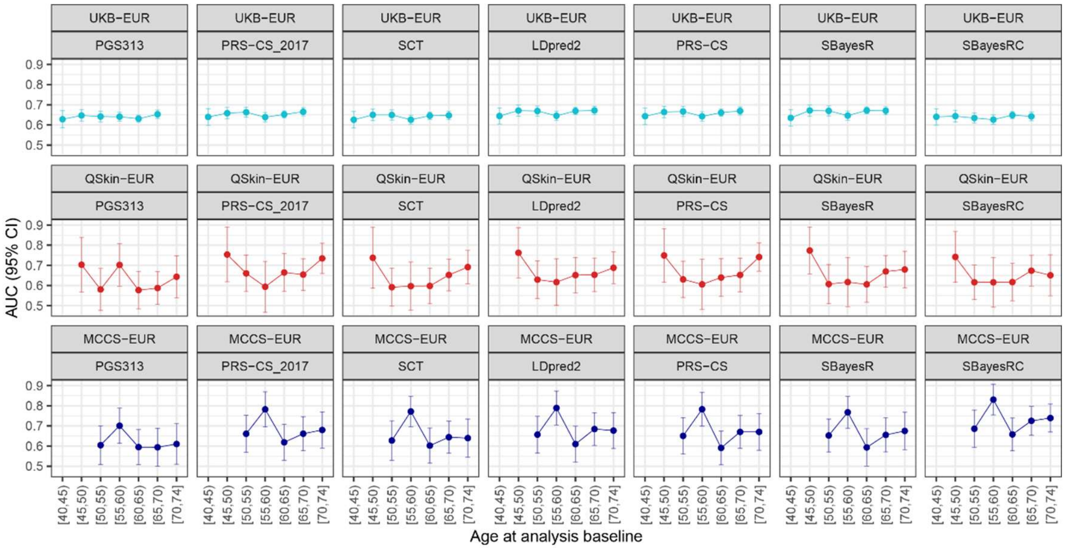

Supplementary Figure 7 - Discrimination results based on PGS Z-scores for European ancestry groups, by age group at baseline. We found consistent predictive performance by age across all three European-ancestry subgroups.

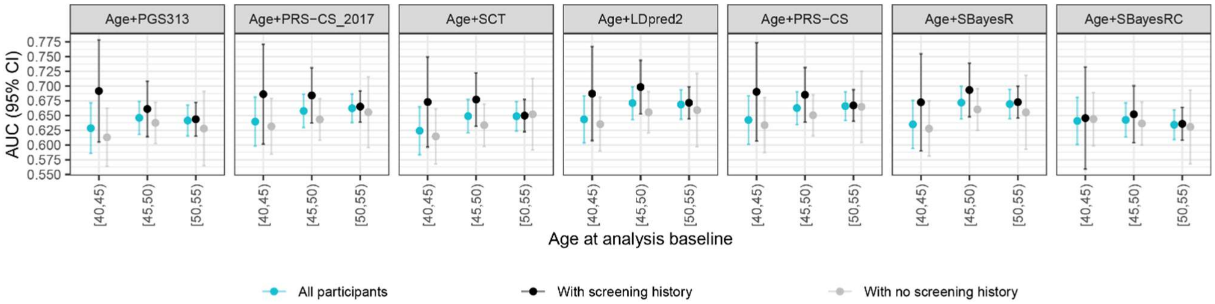

Supplementary Figure 8 - Predictive performance (AUC) of age-PGS-specific absolute 5-year risks, stratified by self-reported pre-baseline breast cancer screening (mammogram) history in the UKB-EUR sub-group. For a given age group, performance of predicted 5-year risks was similar in individuals with and without pre-baseline screening history ($p>0.05$).

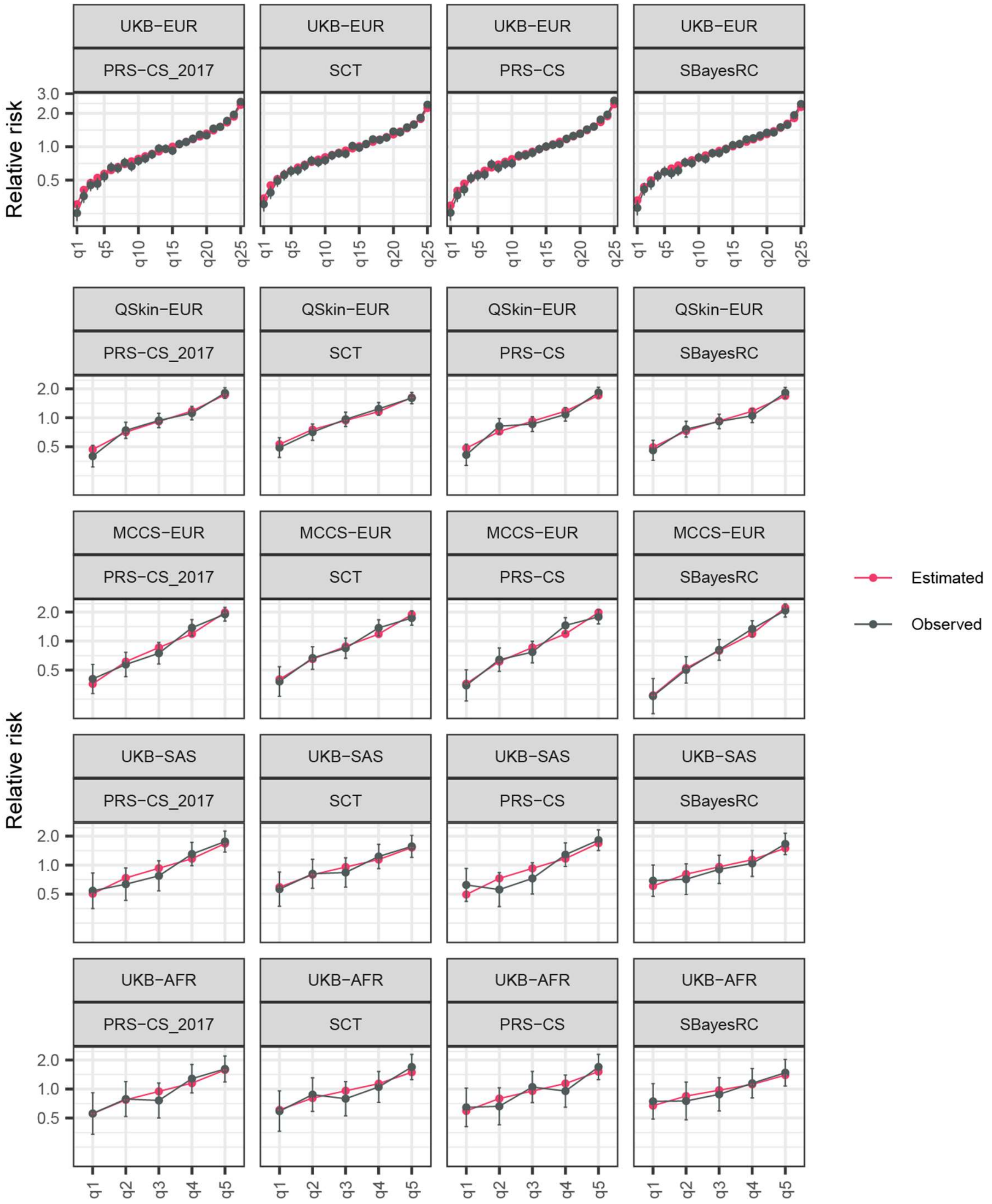

Supplementary Figure 9 - Relative risk for breast cancer diagnoses across PGS Z-score quantiles. The figure presents the relative risk for all breast cancer diagnoses (prevalent and incident) across PGS Z-score quantiles for the cohorts and ancestry subgroups. For UKB-EUR, 25 PGS quantiles were used, with the 13th quantile serving as the reference. For Australian cohorts and UKB ancestry subgroups, five PGS quantiles were used. The red lines represent the estimated relative risk, while the black lines indicate the observed relative risk within each PGS Z-score quantile. Additionally, bars illustrate the 95% confidence intervals around the observed relative risks for each quantile. The plots illustrate the estimated relative risk is well calibrated, as we included cohort- and ancestry-specific AUC, as well as PGS mean and standard deviation used for standardising PGS (calculating PGS Z-scores).

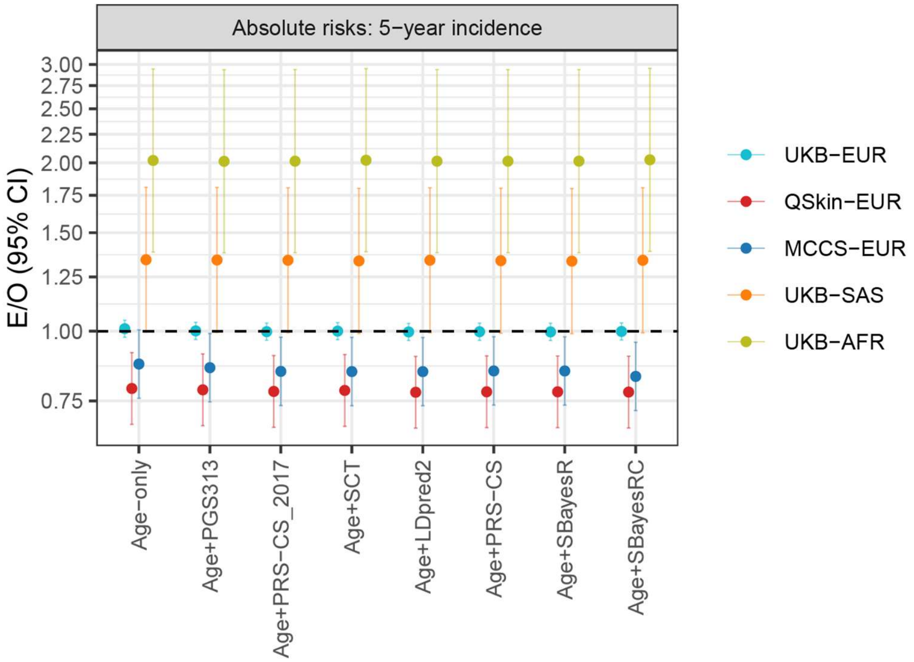

Supplementary Figure 10 - Calibration of expected versus observed 5-year breast cancer incidence across ancestry cohorts. The figure displays the calibration results comparing expected (E; i.e. predicted) to observed (O) 5-year breast cancer incidence across the five ancestry cohorts for 5-year absolute risks. The dashed line at 1 indicates perfect calibration where expected incidence equals observed incidence. The results demonstrate that risk predictions for UKB EUR were well-calibrated, with a calibration close to one. However, predictions underestimated breast cancer incidence in the Australian cohorts and overestimated incidence in the South Asian and African ancestry sub-groups.

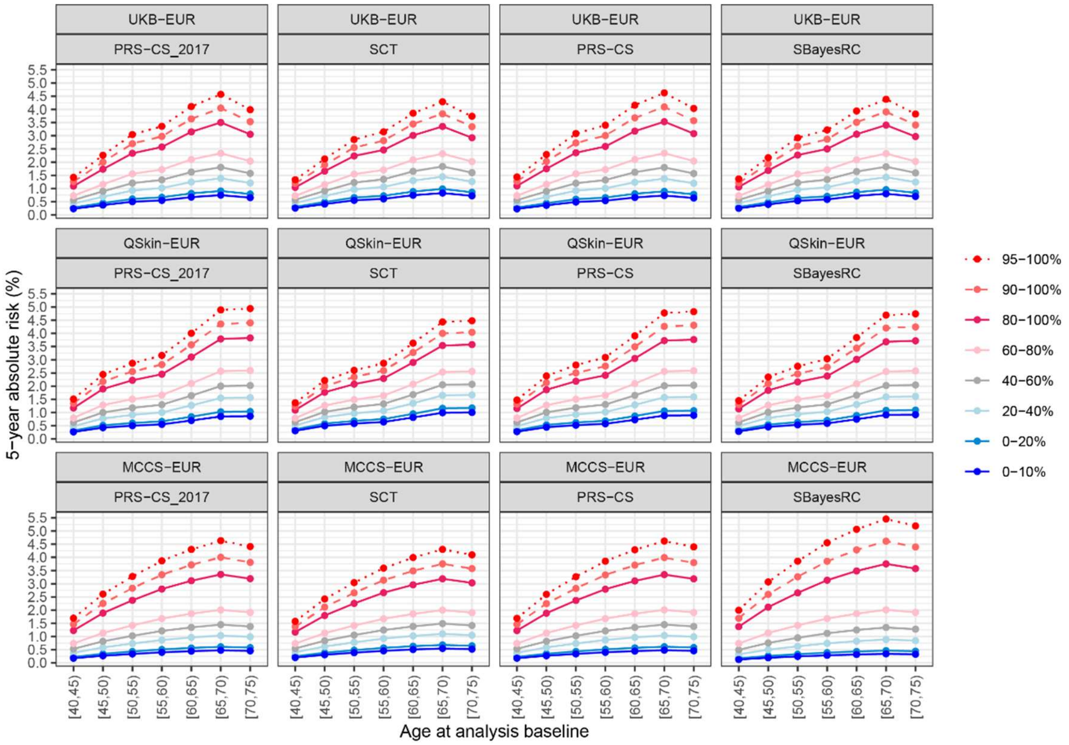

Supplementary Figure 11 - Predicted age-PGS-specific 5-year absolute risk of breast cancer by age and PGS centiles, based on relative risks estimated for participants with inferred European genetic ancestry. This illustrates marked differences between high-risk and low-risk groups at the same age. 5-year absolute risks for the 40-60% quintile (approximately equivalent to population-average risk [i.e., age-specific absolute risk]) are shown in grey.

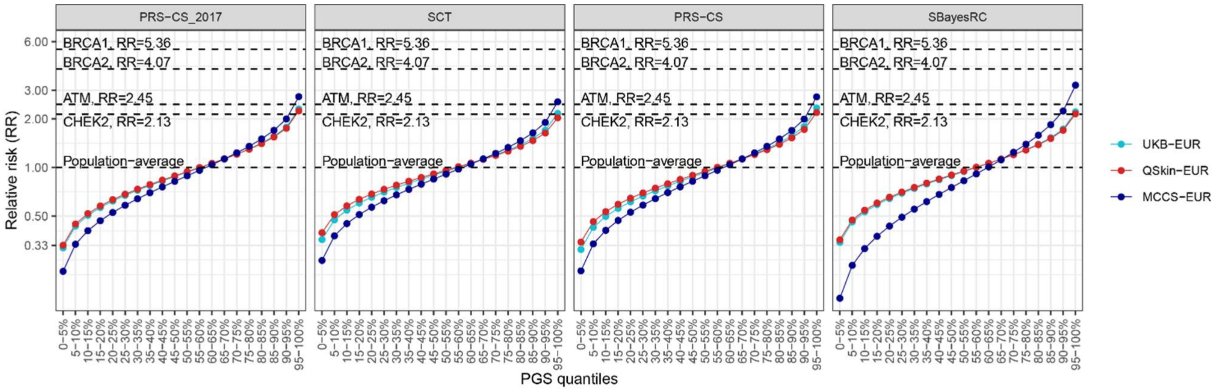

Supplementary Figure 12 – Estimated relative risks of breast cancer conferred by different levels PGS among participants with European genetic ancestry. The relative risks for pathogenic genes were derived from breast cancer case rates reported in the study by Southey et al[26].

3. Supplementary Tables

Supplementary Table 1 - Study cohorts used in discovery GWAS and PGS tuning steps.

	Study cohort				
PGS	UKB-EUR	QSkin-EUR	MCCS-EUR	UKB-SAS	UKB-AFR
**PGS313**	None	None	Discovery GWAS (2017); <2% of GWAS participants	None	None
**PRS-CS$_{2017}$**	~68K Tuning subset (IDs unknown, could not be excluded from evaluations)	None	Discovery GWAS (2017); <2% of GWAS participants	None	None
**SCT**	10K tuning subset (excl. from evaluations)	None	Discovery GWAS (2020); <2% of GWAS participants	None	None
**LDpred2**	10K tuning subset (excl. from evaluations)	None	Discovery GWAS (2020); <2% of GWAS participants	None	None
**PRS-CS**	10K tuning subset (excl. from evaluations)	None	Discovery GWAS (2020); <2% of GWAS participants	None	None
**SBayesR**	None	None	Discovery GWAS (2020); <2% of GWAS participants	None	None
**SBayesRC**	None	None	Discovery GWAS (2020); <2% of GWAS participants	None	None

Supplementary Table 2 - Numbers of incident breast cancers within 5 years of baseline in UKB-EUR, stratified by age and history of breast cancer screening as self-reported at baseline.

	With screening history	Without screening history
Age group	ND / D	ND / D
**[40-45)**	4,312 / 37	15,903 / 143
**[45-50)**	8,671 / 127	19,069 / 239
**[50-55)**	28,225 / 356	5,213 / 64
**[55-60)**	39,168 / 566	530 / 9*
**[60-65)**	51,316 / 909	421 / ≤5*
**[65-70)**	35,708 / ~620^	332 / ≤5*

ND: Number of females not diagnosed with breast cancer during the 5-year follow-up; D: Number of females diagnosed with breast cancer during the 5-year follow-up; * These groups were not considered in the stratified analyses by age as the number of incident breast cancer diagnoses during the 5-year follow-up is <10. Note cell sizes ≤5 were suppressed to preserve confidentiality, where relevant, other cells (denoted by ^) were rounded to ensure the suppressed cells cannot be derived from the remaining information.

Supplementary Table 3 - Number of PGS variants available in each of the study cohorts.

	Number of variants included in PGS	Number of PGS variants available in UKB data	Number of PGS variants available in QSkin data	Number of PGS variants available in MCCS data
**Published PGS**				
**PGS313**	313	305	237*	312
**PRS-CS$_{2017}$**	1,118,831	1,118,822	1,111,267	1,108,437
**Newly generated PGS**				
**SCT**	435,538	435,538	431,116	424,534
**LDpred2**	961,355	961,355	956,071	954,599
**PRS-CS**	941,932	941,932	936,397	935,030
**SBayesR**	789,305	789,305	786,409	787,112
**SBayesRC**	5,387,266	5,387,266	5,341,516	5,284,875

* PGS313 includes 48 insertion/deletion (indel) variants that were not available in the QSkin dataset due to HRC-based imputation.

Supplementary Table 4 – Comparisons of AUCs for GW-PGS Z-scores to AUC for PGS313 Z-scores.

Supplementary Table 5 – Comparisons of AUCs for age-PGS-specific absolute risks to AUC for age-based risks.

Supplementary Table 6 – Comparisons of AUCs for age-PGS-specific risk based on GW-PGS to AUC for age-PGS313-specific risks.

4. Supplementary References

1. Tanha HM, Law MH, Ingold N, Ly P, Olsen CM, Pandeya N *et al*: Polygenic Risk Scores for Prostate Cancer: Comparative Evaluations in UK and Australian Cohorts. *HGG advances* 2025**:** 100477.

2. Smith JL, Schaid DJ, Kullo IJ: Implementing Reporting Standards for Polygenic Risk Scores for Atherosclerotic Cardiovascular Disease. *Current atherosclerosis reports* 2023; **25:** 323-330.

3. Bycroft C, Freeman C, Petkova D, Band G, Elliott LT, Sharp K *et al*: The UK Biobank resource with deep phenotyping and genomic data. *Nature* 2018; **562:** 203-209.

4. Olsen CM, Green AC, Neale RE, Webb PM, Cicero RA, Jackman LM *et al*: Cohort profile: the QSkin Sun and Health Study. *International journal of epidemiology* 2012; **41:** 929-929i.

5. Landi MT, Bishop DT, MacGregor S, Machiela MJ, Stratigos AJ, Ghiorzo P *et al*: Genome-wide association meta-analyses combining multiple risk phenotypes provide insights into the genetic architecture of cutaneous melanoma susceptibility. *Nature genetics* 2020; **52:** 494-504.

6. Milne RL, Fletcher AS, MacInnis RJ, Hodge AM, Hopkins AH, Bassett JK *et al*: Cohort Profile: The Melbourne Collaborative Cohort Study (Health 2020). *International journal of epidemiology* 2017; **46:** 1757-1757i.

7. Manichaikul A, Mychaleckyj JC, Rich SS, Daly K, Sale M, Chen WM: Robust relationship inference in genome-wide association studies. *Bioinformatics (Oxford, England)* 2010; **26:** 2867-2873.

8. Auton A, Brooks LD, Durbin RM, Garrison EP, Kang HM, Korbel JO *et al*: A global reference for human genetic variation. *Nature* 2015; **526:** 68-74.

9. Chang CC, Chow CC, Tellier LC, Vattikuti S, Purcell SM, Lee JJ: Second-generation PLINK: rising to the challenge of larger and richer datasets. *GigaScience* 2015; **4:** 7.

10. Hartigan JA, Wong MA: Algorithm AS 136: A K-Means Clustering Algorithm. *Journal of the Royal Statistical Society Series C (Applied Statistics)* 1979; **28:** 100-108.

11. Mavaddat N, Michailidou K, Dennis J, Lush M, Fachal L, Lee A *et al*: Polygenic Risk Scores for Prediction of Breast Cancer and Breast Cancer Subtypes. *American journal of human genetics* 2019; **104:** 21-34.

12. Fritsche LG, Patil S, Beesley LJ, VandeHaar P, Salvatore M, Ma Y *et al*: Cancer PRSweb: An Online Repository with Polygenic Risk Scores for Major Cancer Traits and Their Evaluation in Two Independent Biobanks. *American journal of human genetics* 2020; **107:** 815-836.

13. Michailidou K, Lindström S, Dennis J, Beesley J, Hui S, Kar S *et al*: Association analysis identifies 65 new breast cancer risk loci. *Nature* 2017; **551:** 92-94.

14. Zhang H, Ahearn TU, Lecarpentier J, Barnes D, Beesley J, Qi G *et al*: Genome-wide association study identifies 32 novel breast cancer susceptibility loci from overall and subtype-specific analyses. *Nature genetics* 2020; **52:** 572-581.

15. Privé F, Vilhjálmsson BJ, Aschard H, Blum MGB: Making the Most of Clumping and Thresholding for Polygenic Scores. *American journal of human genetics* 2019; **105:** 1213-1221.

16. Privé F, Arbel J, Vilhjálmsson BJ: LDpred2: better, faster, stronger. *Bioinformatics (Oxford, England)* 2021; **36:** 5424-5431.

17. Ge T, Chen CY, Ni Y, Feng YA, Smoller JW: Polygenic prediction via Bayesian regression and continuous shrinkage priors. *Nature communications* 2019; **10:** 1776.

18. Lloyd-Jones LR, Zeng J, Sidorenko J, Yengo L, Moser G, Kemper KE *et al*: Improved polygenic prediction by Bayesian multiple regression on summary statistics. *Nature communications* 2019; **10:** 5086.

19. Zheng Z, Liu S, Sidorenko J, Wang Y, Lin T, Yengo L *et al*: Leveraging functional genomic annotations and genome coverage to improve polygenic prediction of complex traits within and between ancestries. *Nature genetics* 2024; **56:** 767-777.

20. Altshuler DM, Gibbs RA, Peltonen L, Altshuler DM, Gibbs RA, Peltonen L *et al*: Integrating common and rare genetic variation in diverse human populations. *Nature* 2010; **467:** 52-58.

21. Gazal S, Finucane HK, Furlotte NA, Loh PR, Palamara PF, Liu X *et al*: Linkage disequilibrium-dependent architecture of human complex traits shows action of negative selection. *Nature genetics* 2017; **49:** 1421-1427.

22. Fay MP, Pfeiffer R, Cronin KA, Le C, Feuer EJ: Age-conditional probabilities of developing cancer. *Statistics in medicine* 2003; **22:** 1837-1848.

23. Pain O, Gillett AC, Austin JC, Folkersen L, Lewis CM: A tool for translating polygenic scores onto the absolute scale using summary statistics. *European journal of human genetics : EJHG* 2022; **30:** 339-348.

24. Li SX, Milne RL, Nguyen-Dumont T, Wang X, English DR, Giles GG *et al*: Prospective Evaluation of the Addition of Polygenic Risk Scores to Breast Cancer Risk Models. *JNCI cancer spectrum* 2021; **5**.

25. Machiela MJ, Chanock SJ: LDlink: a web-based application for exploring population-specific haplotype structure and linking correlated alleles of possible functional variants. *Bioinformatics (Oxford, England)* 2015; **31:** 3555-3557.

26. Southey MC, Dowty JG, Riaz M, Steen JA, Renault AL, Tucker K *et al*: Population-based estimates of breast cancer risk for carriers of pathogenic variants identified by gene-panel testing. *NPJ breast cancer* 2021; **7:** 153.
